# The Use of Procalcitonin (PCT) for Diagnosis of Sepsis in Burn Patients: A Meta-Analysis

**DOI:** 10.1371/journal.pone.0168475

**Published:** 2016-12-22

**Authors:** Luís Cabral, Vera Afreixo, Luís Almeida, José Artur Paiva

**Affiliations:** 1 Department of Plastic Surgery and Burns Unit, Coimbra Hospital and University Centre (CHUC), Coimbra, Portugal; 2 Autonomous Section of Health Sciences (SACS), University of Aveiro, Aveiro, Portugal; 3 CIDMA—Center for Research and Development in Mathematics and Applications; iBiMED—Institute for Biomedicine, University of Aveiro, Aveiro, Portugal; 4 Department of Pharmacology and Therapeutics, Faculty of Medicine, University of Porto, Porto, Portugal; 5 Department of Emergency and Intensive Care Medicine, Centro Hospitalar São João, Porto, Portugal; University of Colorado Denver, UNITED STATES

## Abstract

The continuous development of resuscitation techniques and intensive care reduced the mortality rate induced by the initial shock in burn patients and, currently, infections (especially sepsis) are the main causes of mortality of these patients. The misuse of antimicrobial agents is strongly related to antimicrobial and adverse patient outcomes, development of microbial resistance and increased healthcare-related costs. To overcome these risks, antimicrobial stewardship is mandatory and biomarkers are useful to avoid unnecessary medical prescription, to monitor antimicrobial therapy and to support the decision of its stop. Among a large array of laboratory tests, procalcitonin (PCT) emerged as the leading biomarker to accurately and time-effectively indicate the presence of systemic infection. In the presence of systemic infection, PCT blood levels undergo a sudden and dramatic increase, following the course of the infection, and quickly subside after the control of the septic process. This work is a meta-analysis on PCT performance as a biomarker for sepsis. This meta–analysis showed that overall pooled area under the curve (AUC) is 0.83 (95% CI = 0.76 to 0.90); the estimated cut-off is 1.47 ng/mL. The overall sepsis effect in PCT levels is significant and strong (Cohen's d is 2.1 and 95% CI = 1.1 to 3.2). This meta–analysis showed PCT may be considered as a biomarker with a strong diagnostic ability to discriminate between the septic from the non-septic burn patients. Thus, this work encourages the determination of PCT levels in clinical practice for the management of these patients, in order to timely identify the susceptibility to sepsis and to initiate the antimicrobial therapy, improving the patients’ outcomes.

## Introduction

Comparing to other critical patients, severe burn victims have a higher susceptibility to develop infectious complications leading to sepsis, which is the major cause of mortality in these patients, and may result from intrinsic and extrinsic factors [1,2]. The former may include loss of skin barrier, humoral and cellular immunodepression, presence of necrotic tissue, bacterial translocation and diminution of airway clearance when inhalation injuries are associated. The later comprise the use of invasive devices (intravascular catheters, endotracheal tubes, indwelling bladder catheters, etc.), immobilization and exposition to nosocomial flora [2,3].

Clinical signs and laboratorial findings commonly used to diagnose the presence of infection are not specific and are difficult to interpret due to the magnitude of the systemic inflammatory response unfettered by large burns, which mimics a septic episode. The consensus international definition of sepsis, formulated by the American College of Chest Physicians and by the Society of Critical Care Medicine (ACCP/SCCM) [4,5], was subjected to a revision for burn patients by the American Burn Association [6] (see Annex 1) [S1 File]. This revision implied the modification of some cut-offs and the concomitant documentation of microbiological identification. Nevertheless, currently, the definite identification of microbiological agents still takes two to four days [7]. As stated by Kumar [8] and confirmed by many other studies, the prompt administration of an adequate antimicrobial therapy is the most important isolated factor for the survival of the septic patient and any hourly delay is associated with an increase in mortality.

Burn surgeons are therefore urged to start antimicrobial therapy at the first evidence of infection, but it requires a strong clinical expertise, attending to the lack of a time-effective microbiological confirmation. An adequate therapy reduces the microbial counting, which enables the body systems to control and stop the infectious episode. This is, however, a dynamic process: there are resistant microorganisms that may survive, even when treated with the most effective bactericidal agents. Some microorganisms develop mutations capable of overlapping the antibiotic action, giving rise to microbial resistance (i.e. making the drug ineffective), and thus they may spread to other cells and tissues, being responsible for a systemic infection. To reduce the possibilities of development of microbial resistance is crucial to avoid unnecessary administration of antimicrobial therapy. On the other hand, the prompt beginning of the therapy, with the right dose of an effective drug at the first evidence of infection is equally important, so are the selection of the right drug targeting the microbiological agent and to limit the duration of treatment to the strictly necessary, preventing antibiotic resistance and selective pressure on the microorganisms [9]. This strategy has additional advantages, including the reduction of medication side effects, healthcare-related costs and, in most cases, the length of hospitalization (providing that the surgical treatment of the burns is achieved).

The use of biomarkers has been recommended to help clinicians to timely decide when to start antimicrobial therapy, to monitor its evolution and to advise its early suspension. From the currently available biomarkers, procalcitonin (PCT) has shown the greatest accuracy to indicate the presence of systemic infections within an acceptable timing, in a great range of clinical scenarios [10–12].

This work is an extended and updated version of the paper published by Ren *et al*. [13] [S2 File], including the overall estimation and discussion of several other effect sizes in PCT levels and incorporating four other studies. Its aim is to summarize literature data (through meta-analysis) about the use of PCT for the early detection of sepsis in burn patients, and to discuss the proposed PCT cut-offs for the diagnosis of sepsis.

## Material and Methods

### Data source

PubMed, Scopus and Web of Science databases were used. The combined search term used for this search was: [procalcitonin OR PCT) AND (sepsis OR septic) AND burn]. The search was performed up to 1^st^ December 2015.

### Data extraction, evaluation and synthesis

Only articles written in English focusing on burn patients and on the evaluation of PCT role on the diagnosis and monitoring of septic episodes were considered. Titles and abstracts of records retrieved by the search were screened to determine their relevance. Relevant studies were reviewed in full text, in order to determine their relevance for the meta-analysis. After reading titles and eliminating duplicates (LC and VA), 96 abstracts were independently assessed by three authors (LC, VA and LA), and, from these, 14 references were subjected to detailed analysis and included in the sample, by consensus or majority decision.

### Inclusion and exclusion criteria

A study was considered eligible for inclusion in the meta-analysis if it provided area under the curve (AUC) on serum PCT for diagnosis of sepsis or the serum PCT levels by sepsis and non-sepsis groups in burn patients.

### Statistical analysis

Two techniques were used to calculate the pooled effect estimates: the inverse variance assuming a fixed-effects model, and the DerSimonian-Laird method assuming a random-effects model.

Homogeneity among studies was evaluated using the Cochran’s Q statistic and the I^2^ statistic (the values of 0.25, 0.50, and 0.75 indicating low, moderate, and high degrees of heterogeneity). Publication bias was evaluated using the funnel plot and the Egger regression asymmetry test.

To investigate potentially different effects according to the study, subgroup analyses were performed. Sensitivity analysis to show the impact of each study or subgroup studies on the results was also held.

Meta-DiSc 1.4 (XI Cochrane Colloquium, Barcelona, Spain) was used to calculate the summary receiver operating characteristics (SROC) and the pooled AUC [14]. MetaXL 2.0 (EpiGear International Pty Ltd, Wilston, Queensland, Australia) was used to calculate the pooled Cohen’s d effect sizes (difference of PCT levels between sepsis and non-sepsis groups, the pooled AUC and pooled mean effects [15].

The weight average of all PCT cut-off for sepsis diagnosis proposed in the studies under analysis was also measured.

## Results

**Fig 1:**
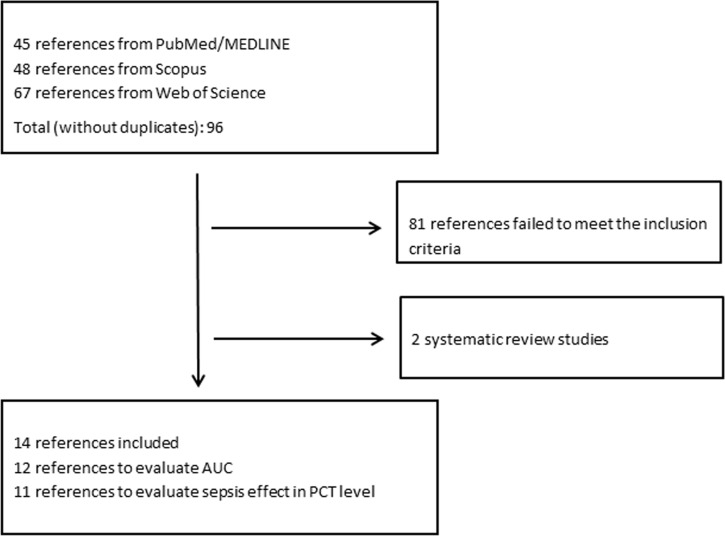
Flow chart for the selection process of studies for evaluation of procalcitonin (PCT) in sepsis diagnosis.

The removal of duplicates from the 160 articles that were initially identified through search in PubMed, Scopus and Web of Science resulted in 96 individual articles (Fig 1). The great majority did not fulfill the eligibility criteria. After exclusion of ineligible papers, 14 articles, comprising a temporal range from 1997 to 2015, were found to meet the inclusion criteria and were selected for review.

Plasma PCT concentrations had been measured using different methods, such as PCT-Q immunochromatography (Brahms Diagnostica, Berlin, Germany) [15,16], PCT-Lumi immunoluminometric (Brahms Diagnostica, Berlin, Germany) [16–21], electrochemical luminescence immunoassay (ECLIA) (Brahms Diagnostica, Berlin, Germany) [22–24], and immunoassay sandwich and final fluorescence technique (VIDAS, bioMérieux, Marcy L’Etoile, France) [25].

Two studies were pediatric [20,26], one mixed [18] and the remaining studies included only adult patients [15–19,21,23–25,27–29]^.^

**Fig 2:**
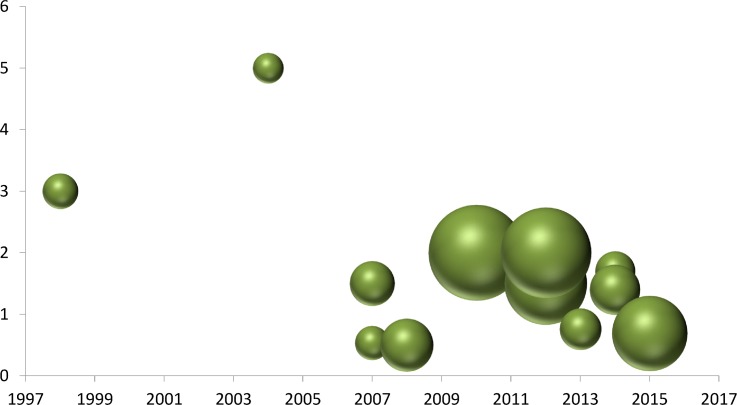
Bubble plot of cut-off for procalcitonin (PCT) in sepsis diagnosis for 12 studies organized by year. Bubble size corresponds to the number of time-points.

Studies also differ in the PCT cut-off defined for sepsis suspicion. Reported cut-off values include 0.5 ng/mL [23], 0.534 ng/mL [16], 0.69 ng/mL [22], 1.5 ng/mL [19,27], 1.7 ng/ mL [23], increment of 1.5 ng/mL in two consecutive days [18], 2 ng/mL [25,29]; 3 ng/mL [17], and 5 ng/mL [20]. Using the different cut-offs for sepsis diagnosis proposed in each study (Table 1), the weight average of all PCT cut-offs for sepsis was computed and the resulting cut-off was 1.59 ng/mL. Fig 2 shows a bubble plot of cut-off for PCT in sepsis diagnosis for 12 studies organized by year. The two older studies showed the highest cut-offs values; if these two studies are excluded, the estimated value is 1.47 ng/mL.

**Table 1:** Area under the curve (AUC) and the corresponding standard error (SE) for each study evaluating the ablity of procalcitonin (PCT) as a biomarker, and the overall estimate using random effects model.

Study	Cut-off (ng/mL)	Time points	N	ROC AUC	SE	95%CI	Tp	Fp	Fn	Tn
Sachse, 1999 (	N/A	N/A	19	N/A	N/A	N/A	N/A	N/A	N/A	N/A
von Heimburg, 1998	3	27	27	N/A	N/A	N/A	2	0	16	9
Neely, 2004	5	62	20	N/A	N/A	N/A	11	12	15	24
Abdel-Hafez 2007	N/A	N/A	42	N/A	N/A	N/A	N/A	N/A	N/A	N/A
Bargues, 2007	0.534	359	25	0.66	0.04	0.59–0.72	39	29	53	237
Lavrentieva, 2007	1.5	934	43	0.98	0.03	0.91–1.04	93	72	21	748
Barati, 2008	0.5	60	60	0.97	0.02	0.93–1.01	30	3	0	27
Bognar, 2010	2	196	28	0.77	0.03	0.70–0.83	73	32	11	78
Lavrentieva, 2012	1.5	139	145	0.97	0.01	0.94–0.99	64	5	9	67
Kim, 2012	2	175	175	0.84	0.03	0.79–0.90	72	15	21	67
Cakir Madenci, 2013	0.759	611	37	0.85	0.02	0.81–0.88	181	79	59	292
Seoane, 2014	1.7	34	34	0.55	0.11	0.33–0.77	4	0	12	18
Paratz, 2014	1.4	345	54	0.62	0.04	0.54–0.70	38	190	10	106
Mokline, 2015	0.69	121	121	0.93	0.03	0.87–0.98	39	12	5	65
Total (AUC random effects)				0.83	0.04	0.76–0.90				
Q				182.0						
p-value				<0.001						
I^2^				95%						

N—total number of individuals; ROC AUC—receiver operating characteristic area under the curve; 95%CI—95% confidence interval; Fn—false negative; Fp—false positive; N/A–not available; Tn—true negative; Tp—true positive.

### Data uniformization

Data uniformization is required for the meta-analysis of Cohen’s d effect size. In the study of Sachse *et al*. [18], PCT values were reported as the median by different post-burn time intervals (6 distinct intervals) for septic and non-septic groups; the average and the standard deviation of PCT values were deduced assuming the normal behavior of PCT values ($mean=\frac{\sum {median}_{i}}{6};\;std={\sqrt{n}\;std}_{median}$).

In Neely *et al*. [20] and Lavrentieva *et al*. [27] (both in sepsis and non-sepsis groups PCT standard deviation by each group was obtained using the inter-quartile distance and assuming the normal behavior of PCT values (interquartile range = 1.35σ). In Cakir Madenci *et al*. paper [22], it was calculated using the quantiles 2.5% and 97.5% and the normality assumption (x_0.975_-x_0.025_ = 3.92σ).

In Bargues *et al*. [16], log values were transformed and combined in order to obtain the PCT average and standard deviation, and the subgroup values are combined to obtain the pooled standard deviation and the weight average. Lavrentieva *et al*. [19] combined subgroup values to obtain the pooled standard deviation and the weight average of PCT for non-sepsis groups.

As Barati *et al*. [15], Bognar *et al*. [29] and Mokline *et al*. [21] did not present AUC standard error, the estimates for standard error were computed using the Hanley and McNeil procedure [30]. Paratz *et al*. [24] reported the PCT discriminative power as not significant with AUC = 0.38 (95% Confidence Interval (95% CI) 0.29 to 0.46). However, if the classifier was negated on every instance, the true positive (TP) classifications become false negative and the false positive become true negative (TN), and we obtain AUC = 0.62 and, for the cut-off chosen by the authors (1.4 ng/mL), the corresponding sensitivity is 80 and the specificity is 36.

In Lavrentieva *et al*. [27], Kim *et al*. [25], Cakir *et al*. [22], Seoane *et al*. [23] and Paratz *et al*. [24], the standard error is estimated from AUC confidence interval (SE = (UB-LB) / 3.92). Table 1 presents the estimate AUC and the corresponding standard error for each study.

To evaluate PCT as a diagnostic marker for sepsis, there are several studies based on timepoints with repeated measures and others with independent ones and, in this context, the results were used as independent.

## Meta-analysis

For all effect sizes under analysis, the studies show significant heterogeneity (p<0.01, I^2^>50%), thus a random-effects model for meta-analysis was used.

AUC plays a central role in evaluating diagnostic ability of tests, in particular of PCT biomarker. Ten studies under analysis present the PCT AUC estimate value and the first four studies reported in Table 1 do not present AUC values. Table 1 presents the overall estimated AUC for PCT for sepsis diagnosis, where the pooled estimate is 0.83 (95% CI = 0.76 to 0.90). PCT diagnosis ability is significant (AUC>0.5) and the effect size is strong.

**Fig 3:**
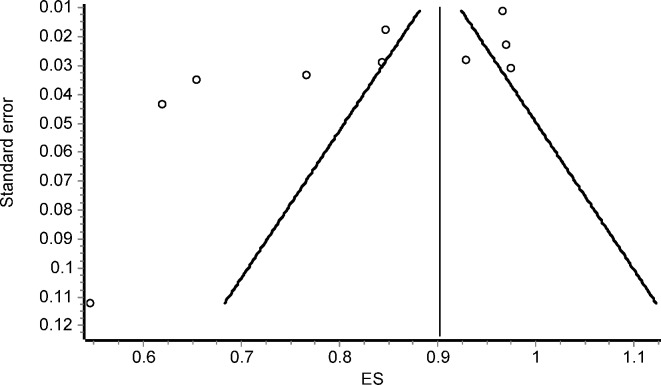
Funnel plot of the AUC on diagnostic sepsis effect

The publication bias associated with the AUC on diagnostic sepsis effect was analysed by the funnel plot and the Egger test. The result of Egger's test was significant (p <0.001), which is manisfested in funnel plot asymmetry (Fig 3). It is of note that the studies appearing to have higher effect in the publication bias are those which had lower AUC values.

To find out sources of heterogeneity, a subgroup analysis was done, using the random effect model, according to different criteria used for sepsis determination in the works of the sample, namely clinical evaluation, Baltimore Sepsis Scale, American College of Chest Physicians/Society of Critical Care Medicine (ACCP/SCCM) definition and the more recent and specific one from the American Burn Association (ABA) (Table 2). In order to reduce subjectivity using standardized concepts, this analysis included just the works explicitly employing the ACCP/CSCCM or the ABA definition. For the former subgroup, the AUC was 0.87 (95% CI = 0.63 to 1.0) and for the later it was 0.87 (95% CI = 0.71 to 0.90).

We also conducted another subgroup analysis, excluding retrospective studies [31,32], achieved an AUC of 0.86 (95% CI = 0.78 to 0.93).

**Table 2:** Study characterization by sepsis criteria employed, type of design and population age.

Study	Sepsis Criteria	Design Type	Population Age
von Heimburg, 1998	BSS	Prospective	Adult
Sasche, 1999	Clinical	Retrospective	Mixed
Neely, 2004	Clinical	Prospective	Paediatric
Abdel-Hafez, 2007	Clinical	Prospective	Paediatric
Bargues, 2007	ACCP/SCCM	Prospective	Adult
Lavrentieva, 2007	ACCP/SCCM	Prospective	Adult
Barati, 2008	ACCP/SCCM	Prospective	Adult
Bognar, 2010	ABA	Prospective	Adult
Lavrentieva, 2012	ABA	Prospective	Adult
Kim, 2012	Clinical	Prospective	Adult
Cakir Madenci, 2013	ABA	Prospective	Adult
Seoane, 2014	ACCP/SCCM	Retrospective	Adult
Paratz, 2014	ABA	Prospective	Adult
Mokline, 2015	ACCP/SCCM	Prospective	Adult

Similarly to the analysis presented by Ren *et al*. [13], the summary receiver operating characteristic (SROC) for PCT in sepsis diagnosis was obtained including all the studies considered (four additional studies to those included in Ren *et al*.). Data reported for SROC estimation by these authors have, however, some differences in comparison to the data used in the present work (Table 1). When the study reported the use of several time-points, the total of time-points was used as sample size instead of the total number of individuals [16,19,21,22,24,29]. Moreover, as the revision from Bognar *et al*. [29] was developed including only septic patients, this feature could add some additional bias.

**Fig 4:**
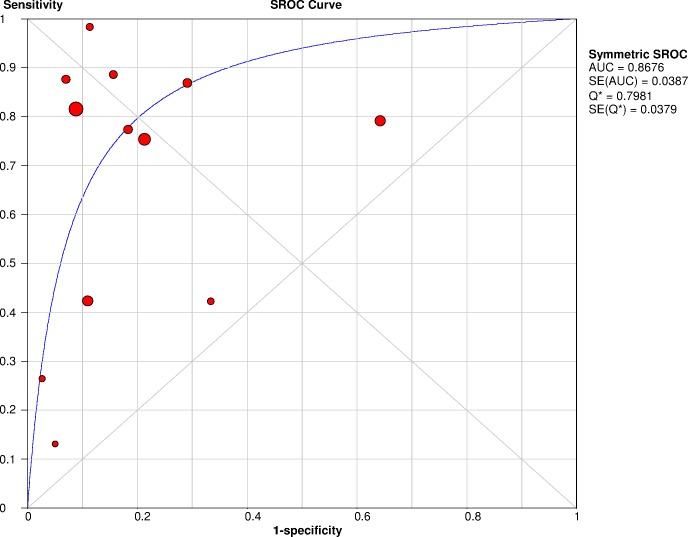
Summary receiver operating characteristic (SROC) curve of procalcitonin (PCT) for the diagnosis of sepsis in burn patients.

Fig 4 plots the sensitivity vs the false positive rate of all studies (using the values indicated in Table 1), presenting the SROC and achieving an overall AUC of 0.87 (SE = 0.04). The results produced by this method are in accordance with those obtained directly by the DerSimonian-Laird method (Table 1). The pooled sensitivity and specificity are 0.77 (95% CI = 0.72 to 0.80) and 0.65 (95% CI = 0.62 to 0.69), respectively.

PCT mean values for sepsis and non-sepsis groups for eleven individual studies are presented in Table 3. All the studies presenting the values by groups were considered. Due to the significant heterogeneity, the overall mean estimate was obtained assuming the random effects model: 46.8 ng/mL (95%CI = 2.5 to 91.1) for sepsis group and 0.9 ng/mL (95%CI = 0.1 to 1.6) for non-sepsis group. This analysis is useful to evaluate the strength of the PCT concentration for each group, and a statistically significant mean difference was observed between sepsis and non-sepsis groups.

**Table 3:** Procalcitonin (PCT) mean values and the corresponding standard error (SE) for each study, and the overall estimate using random effects model estimated by group (sepsis and non-sepsis group).

	Sepsis group			Non-sepsis group		
Study and year	Mean	SE	N	Mean	SE	N
Sachse, 1999	3.9	11.7	9	0.4	0.4	10
von Heimburg, 1998	49.8	76.9	18	2.3	3.8	9
Neely, 2004	6.7	20.4	36	2.1	3.2	26
Abdel-Hafez, 2007	369.1	11.4	20	47.4	10.7	22
Bargues, 2007	45.5	10.9	92	2.8	1.1	267
Lavrentieva, 2007	11.8	15.8	114	0.6	0.4	820
Barati, 2008	8.5	7.8	30	0.5	1.0	30
Lavrentieva, 2012	7.2	24.1	86	0.7	2.8	53
Cakir Madenci, 2013	2.0	22.0	240	0.3	2.7	371
Seoane, 2014	3.0	5.4	16	0.6	0.3	18
Mokline, 2015	7.3	7.0	44	0.9	0.5	77
Total (random effects)	46.8	22.6		0.9	0.4	
Q	19649			24		
p-value	<0.001			0.004		
I^2^	100%			63%		
95%CI	2.49–91.05			0.10–1.61		

N—total number of individuals; 95%CI—95% confidence interval; SE—standard error.

In the sepsis group, there are three studies with very high PCT concentration (von Heimburg *et al*. [17], Bargues *et al*. [16], Abdel-Hafez *et al*. [26]) (>45 ng/mL); excluding these studies, an overall PCT mean value of 6.4 ng/mL (95%CI = 3.8 to 9.0) was obtained for the sepsis group and of 0.6 ng/mL (95%CI 0.2 to 0.9) for the non-sepsis group. The mean results are robust after the exclusion of these three studies, which perhaps shall be considered as outliers related to different dosing methodology.

**Fig 5:**
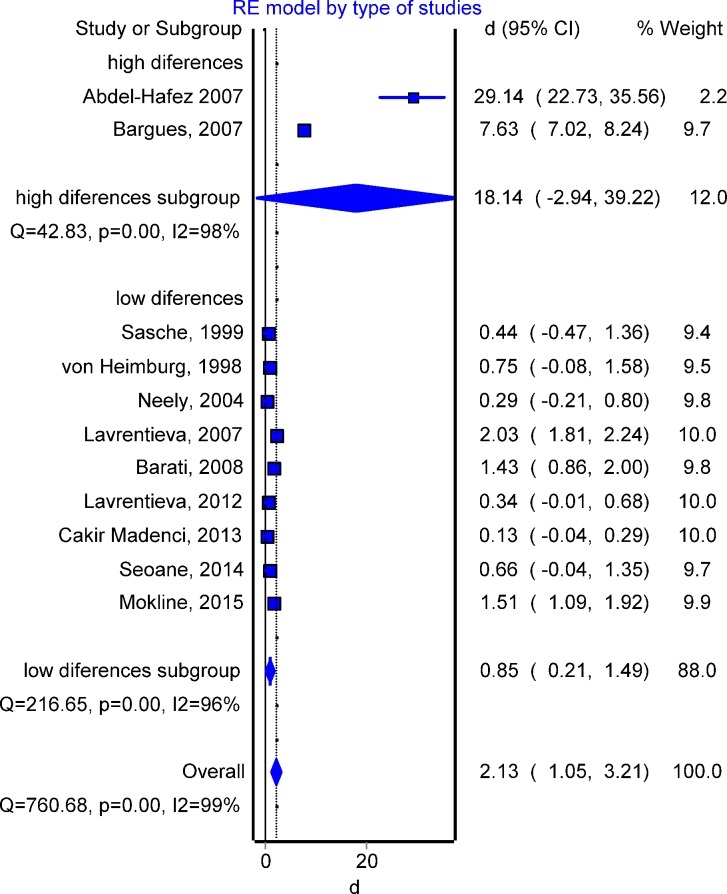
Forest plot for sepsis effect on procalcitonin (PCT) concentration. The estimated overall effect size and confidence interval (Cohen’s d, displayed as a diamond) and individual effect sizes (Cohen’s d, displayed as a rectangle) are shown.

Fig 5 shows the mean difference effect sizes (sepsis and non-sepsis group on PCT concentration) for the eleven studies. Two of these studies (Abdel-Hafez e*t al*. [26], Bargues *et al*. [16]) reported a much higher difference between groups than the difference observed in the other studies. Due to this clear heterogeneity, a subgroup analysis was performed. Inside both subgroups, the heterogeneity is also significant (p<0.001, Cochrane Q test), justifying therefore the use of random effects models. The overall sepsis effect is significant (95%CI = 1.1 to 3.2 with overall estimate of 2.1 ng/mL). Including only the low difference group, the overall effect remains significant, but the effect strength is lower (0.9 ng/mL with 95%CI = 0.2 to 1.5), as expected.

Sensitivity analysis by excluding one study at each turn and pooling results from the remainder further confirmed the robustness of the findings, confirming the significance of the sepsis effect on PCT concentration (Table 4).

**Table 4:** Sensitivity analysis of overall sepsis effect (Cohen’s d) in procalcitonin (PCT) levels in burn patients.

Excluded study	Pooled d	95%CI
Sachse, 1999	2.317	1.170–3.464
von Heimburg, 1998	2.290	1.139–3.442
Neely, 2004	2.365	1.190–3.539
Abdel-Hafez 2007	1.520	0.480–2.559
Bargues, 2007	1.182	0.447–1.917
Lavrentieva, 2007	2.279	0.992–3.566
Barati, 2008	2.237	1.067–3.406
Lavrentieva, 2012	2.404	1.183–3.624
Cakir Madenci, 2013	2.467	1.204–3.730
Seoane, 2014	2.309	1.150–3.468
Mokline, 2015	2.255	1.057–3.453

95%CI–95% confidence interval.

**Fig 6:**
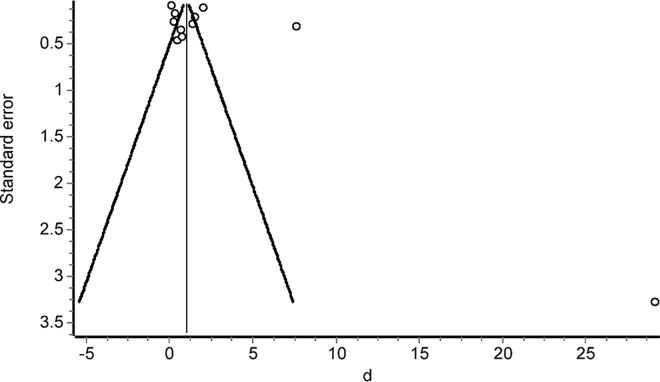
Funnel plot of the difference of procalcitonin (PCT) levels between sepsis and non-sepsis groups (Cohen’s d effect sizes).

The result of Egger's test was not significant (p = 0.194). Thus the publication bias associated to the meta-analysis of difference of PCT levels between sepsis and non-sepsis groups seems to be not relevant. However, the oldest studies included in the meta-analysis of sepsis effect (Fig 5) caused (non-significant) funnel plot asymmetry (Fig 6). Doing again a subgroup analysis based on the used sepsis definition, the resulting values for Cohen’s d were 3.69 (95% CI = 0.45 to 6.92) when ACCP/SCCM classification was employed; 0.64 (95% CI = 0.02 to 1.26) according to ABA classification and 3.38 for the rest (95% CI = 0.90 to 5.87).

## Discussion

Burns represent a public health problem and are an important cause of mortality and morbidity around the World. According to the World Health Organization (WHO), it is estimated that 265 000 deaths occur every year from fire-related burn injuries. Most of these injuries occur in low- and middle-income countries and almost half of these cases are registered in the South-East Asia Region. Moreover, burns are one of the major causes of disability-adjusted life years (DALYs) lost in these countries. It was estimated in 2004 that nearly 11 million people worldwide were burned severely enough to require medical support. Burns also significantly impact the healthcare-related costs, in particular concerning prolonged hospitalization periods, burn management, and care for disfigurement and emotional trauma [33].

Severe burn injuries that affect all body systems and their regulatory pathways may be considered as a paradigm of polytrauma. Tissue injury, coupled with the release of multiple local and systemic mediators of inflammation, leads to an increase in vascular permeability, resulting in marked hydroelectrolytic and cardiovascular alterations [34]. These alterations rapidly evolve to a state of hypovolemic shock, with loss of water, proteins and electrolytes, which is usually fatal if not adequately treated. In the past, shock was indeed the first cause of death in these patients. However, the great advances observed in intensive care have reversed this situation and today this initial acute phase of hypovolemia is overcome with success in the majority of the cases [35]. Nowadays, sepsis has become the major cause of death in burn patients, occurring generally in a late post-traumatic period [35,36].

Considering the patients with suspected infection, septic patients have obviously the worst outcomes [37]. These outcomes may be highly improved, if the appropriate antibiotics are administered early and timely [38]. The use of reliable biomarkers that early identify a septic process may have a great importance to help the physicians to select patients for prompt antibiotic therapy, particularly when clinical signs are absent or unclear. On the other hand, if the biomarker levels are under the cut-off values defined for septic processes, this information may suggest that an inflammatory non-infectious process is occurring. So, these tests are also useful to avoid unnecessary antibiotherapy, which may result in toxicity and development of antimicrobial resistance.

PCT is a 116-aminoacid prohormone of calcitonin, which is mainly produced by the C-cells of thyroid gland and participates in calcium metabolism [39]. PCT is also synthesised in other tissues, including liver, kidney, lung and adipose tissue, in response to endotoxins, cytokines and other mediators released during the infection period [40]. PCT blood levels are barely detectable in healthy individuals. However, in the presence of systemic bacterial infection or, in a lower scale, fungal infection, its levels suddenly undergo a dramatic increase, following the infection course and then quickly subside after the control of the septic process. There is strong clinical evidence that PCT allows differentiation between non-infectious systemic inflammatory response and microbiological infection by bacteria or fungi and several studies confirm its utility as a reliable means to guide antibiotic use in community-acquired pneumonia and sepsis in intensive care patients [11,12,41–46]. Some studies also suggest its usefulness in the diagnosis and prognosis of sepsis in burns patients [17–19,27,47] [S3 File], though some controversy still persists [16,28,48]. PCT is currently one of the most investigated biomarkers and has already been integrated in treatment algorithms for patients with lower respiratory airways infections [12] and for ICU patients [28].

The main finding of this meta-analysis is that most of the included studies indicate that PCT can be a simple and very useful biomarker for the early identification of sepsis in burn patients, when used in combination with relevant clinical examination and other biomarkers available (e.g. leukocytosis, C-reactive protein, MR-pro-adrenomedullin) [18,49–52]. In fact, the pooled information resulting from this work suggests the feasibility of PCT quantification in these patients, showing that an average cut-off of 1.5 ng/mL is a strong indicator for sepsis suspicion and therefore for the initiation of antibiotherapy.

In addition, this work demonstrated that overall pooled area under the SROC curve was 0.87, with a sensitivity of 0.77 and a specificity of 0.65. The area under the SROC curve and the sensitivity are in agreement with the results published by Ren *et al*. [13], which reported that the area under the SROC curve was 0.92, with a sensitivity of 0.74. On the other hand, the specificity reported in this work was lower (0.65 vs 0.88) and the publication bias was significant. Thus, the inclusion of four additional studies in this meta-analysis, including two pediatric studies, contribute to support and strengthen the evidence supporting the interest of PCT levels as a biomarker for the early diagnosis of sepsis in burn patients. The sensitivity analysis, performed by excluding one study at each turn, also confirmed the sepsis effect on PCT concentration in burn patients. Nevertheless, the inclusion of these pediatric studies may explain the lower specificity and the higher heterogeneity reported in our work, as a population with different physiologic characteristics was considered in the analysis.

This work also included a sub-group analysis, comparing sepsis and non-sepsis groups of the studies included. This analysis revealed that sepsis group showed a statistical significant increase in the PCT mean values, in comparison with non-sepsis group. It also indicated that both groups were highly heterogeneous, though this parameter was higher in the sepsis group. Moreover, no significant publication bias was registered between sepsis and non-sepsis groups. The increase of PCT levels in patients diagnosed with sepsis corroborates the potential usefulness of this prohormone in burn patients with sepsis.

However, some studies reported that PCT levels can temporarily increase in some patients postoperatively, even in the absence of infection [41]. This increase is minor and rapidly subsides, but it must obviously be taken into account. In addition, some previous studies did not confirm that PCT levels may be helpful for the diagnosis of sepsis in burn patients [23,24,48], which may result from several factors, such as a small sample size, heterogeneity among patients included in the analysis, different criteria for sepsis diagnosis and different timings of sampling [25].

This work has some limitations that must be considered when interpreting the results. Only 12 studies were available for meta-analysis and the number of patients included was in general small and heterogeneous between the different studies. The cut-offs values, which ranged from 0.5 to 5 ng/mL, and the methods used to quantify the PCT concentration also diverged in these studies. The high heterogeneity of the studies is also a factor that may rise questions about the utility of this biomarker. The inclusion of two pediatric studies, as referred, may had a significant impact in this parameter. Another limitation relies on the origin of the data included in the analysis. In fact, only published studies written in English were considered, which may imply the exclusion of significant and important data obtained in unpublished studies and studies written in other languages.

Based on these studies, in the authors’ opinion, PCT levels should be determined daily in burn patients at high risk of infection (large total body surface area [TBSA] burns, mechanical ventilation, comorbidities, etc.), and at least twice a week for the rest of the burn patients. However, further studies with significant number of patients and planned to reduce the variability of cut-off values, number of timepoints and methods to quantify PCT levels should be conducted, to better evaluate the interest of PCT as a biomarker for early diagnosis of sepsis in burn patients. Studies combining the determination of PCT levels and the evaluation of other potential biomarkers or other clinical evidence should also be done, as generally the single determination of one biomarker is not sufficient to predict or early diagnose the septic process.

## Conclusion

This meta–analysis showed PCT may be considered as a biomarker with a strong diagnostic ability to discriminate between the septic and the non-septic burn patients. The overall sepsis effect is significant and the overall association between PCT levels and the occurrence of mortality is also significant. This work clearly encourages the serial and frequent measurement of PCT levels in clinical practice for the management of burn patients, in order to timely identify the susceptibility to sepsis and to initiate the antimicrobial therapy, improving the patients’ outcomes

## Supporting Information

S1 File This is the document in Annex 1. (DOCX)

S2 File This is the file of Systematic Revision–H. Rao.
(PDF)

S3 File This is the file of Systematic Revision–E. Mann.
(PDF)

S4 File This is the file of PRISMA 2009 checklist—The Use of Procalcitonin (PCT) for Diagnosis of Sepsis in Burn Patients. (DOC)
