# Supplementary material for: The Use of Procalcitonin (PCT) for Diagnosis of Sepsis in Burn Patients: A Meta-Analysis
Source: PLoS One. 2016 Dec 22;11(12):e0168475. doi: 10.1371/journal.pone.0168475 (PMC5179235; doi:10.1371/journal.pone.0168475)
Supplement: S1 File — (DOCX) [file pone.0168475.s001.docx]

**Annex I**

**Criteria for Sepsis Identification in Adult Patients**

| **Signs and Lab Exams** | **ACCP/SCCM (⇒ ≥ 2)** | **ABA (⇒ ≥ 3)** |
| --- | --- | --- |
| Temperature | > 38 ºC or < 36ºC | > 39 ºC or < 36.5 ºC |
| Heart Rate | > 90/min | > 110/min |
| Respiratory Rate | > 20/min or pCO_2_ < 32 mmHg | > 25/min or need resp. vol.> 12L/min for ventilated pts. |
| Leucocytes Counting | > 12,000/mm^3^ or  < 4,000/mm^3^ or  > 10% immature forms | > 12,000/mm^3^ or  < 4,000/mm^3^ or  > 10% immature forms |
| Arterial Pressure |  | Refractory hypertension with:  systolic art. pressure < 90 mmHg or  medium art. pressure < 70 mmHg or  ↓ medium art. pressure < 40 mmHg |
| Platelets Counting |  | < 100,000/μL |
| Glycemia |  | Blood glucosis > 200 mg/dL (7,7mM/L) in the absence of diabetes |
| Enteral Feeding Intolerance |  | Inability to continue enteral feedings > 24 h (abdominal distension or high gastric residuals, residuals two times feeding rate or uncontrollable diarrhea, > 2500 mL/day) |
| **+** | | |
| **Confirmed Evidence of Infection:**   - Invasion of deep tissues   (> 10^5^ bacterial colonies/g or evidence of vascular invasion in burn biopsy) or     - Bacteremia/Fungemia or - Documented Infection (RX, CAT, MRI) | | |
