# Supplementary material for: The Use of Procalcitonin (PCT) for Diagnosis of Sepsis in Burn Patients: A Meta-Analysis
Source: PLoS One. 2016 Dec 22;11(12):e0168475. doi: 10.1371/journal.pone.0168475 (PMC5179235; doi:10.1371/journal.pone.0168475)
Supplement: S2 File — Rao. (PDF) [file pone.0168475.s002.pdf]

Available online at www.sciencedirect.com

ScienceDirect

journal homepage: www.elsevier.com/locate/burns

Serum procalcitonin as a diagnostic biomarker for sepsis in burned patients: A meta-analysis

Haitao Ren [a], *Yuan Li* [b,*], *Chunmao Han* [a], *Hang Hu* [a]

[a] *Department of Burns and Wound Center, The Second Affiliated Hospital, School of Medicine, Zhejiang University, Hangzhou 310009, China*
[b] *Department of Ultrasound, Women's Hospital, School of Medicine, Zhejiang University, Hangzhou 310006, China*

ARTICLE INFO

Article history:
Accepted 25 August 2014

Keywords:
Biological markers
Burn
Diagnostic tests
Sepsis
Procalcitonin

ABSTRACT

Background: Sepsis is one of the main causes of mortality in severe burns. However, it is difficult to diagnose early. Procalcitonin (PCT) has been reported as a biomarker for sepsis with controversial results. The aim of the study is to assess the diagnostic value of serum PCT for sepsis in burn patients through a meta-analysis of published studies.
Methods: A comprehensive literature search of PubMed, Embase, Web of Science and the Cochrane Library databases for studies published up to 1st March 2014 that evaluated PCT as a marker for diagnosing sepsis in burn patients was conducted. The summary receiver operating characteristic curves served to evaulate overall test performance. Meta-Disc 1.4 software and Stata 12.1 were used to analyze the data.
Results: A total of 566 patients (samples) from nine trials were identified and analyzed. The pooled sensitivity and specificity were 0.74 and 0.88, respectively. No threshold effect was found among studies. The area under the SROC curve (AUC) was 0.92.
Conclusion: The results suggest that serum PCT is a useful biomarker (AUC = 0.92) for early diagnosis of sepsis in burn patients. However, the results should be used with caution, because of obvious heterogeneity among those studies. Further large-scale research should regard more attention to the uniform cut-off value, and laboratories test methods.
© 2014 Elsevier Ltd and ISBI. All rights reserved.

1. Introduction

Sepsis is an inflammatory response to severe infection with the existence of organ dysfunction [1,2] and is also one of the principal reasons of mortality in burned patients [3–7]. It is important but difficult to diagnose sepsis early and accurately [8–11]. However, some patients with sepsis have the similar symptoms as those with non-infectious causes of SIRS [12]. Blood microbiological cultures can help the identification of systematic bacterial infection, but the results often reported late and yield false positive or negative results [13,14]. Traditional markers such as CRP (C-reactive protein) and WBC (white blood cell) are too weak to accurately identify sepsis in the burned patient, because of the baseline inflammatory response [15] and immunopathies [16].

The 116-aminoacid polypeptide procalcitonin (PCT) has been studied as a biomarker for sepsis. PCT assay has been used to detect sepsis in critically ill patients and showed promising in differentiating sepsis from other non-sepsis situations [17–27]. Procalcitonin is also elevated in certain conditions such as major burns, trauma, surgery, and multiorgan failure as well as

* *Corresponding author*. Tel.: +86 13777822681.
E-mail address: Docliyuan@126.com (Y. Li).
http://dx.doi.org/10.1016/j.burns.2014.08.019
0305-4179/© 2014 Elsevier Ltd and ISBI. All rights reserved.

infection [28–30]. However, more recent studies have produced conflicting results [31–38] and the studies performed in the burned patients showed a diverse sensitivity and specificity [20,39–47]. Mann [29] systematically reviewed the effect of PCT in diagnosing sepsis in burn patients in 2011. Since then, some new larger-scale studies of procalcitonin or with different designs have been done and our understanding of procalcitonin is still developing.

It is necessary to assess the value of serum PCT for the diagnosis of sepsis in burned patients through a meta-analysis of published studies. Based on Mann's reviews [29] and other relative studies, we performed this meta-analysis.

2. Methods

2.1. *Articles retrieval and search strategy*

Four reviewers systematically reviewed PubMed, EMBASE, Web of Science, and the Cochrane Library databases up to 1st March 2014. The PubMed combined search term used was: (procalcitonin OR PCT) AND (sepsis) AND burn. The search strategy was designed for each database.

2.2. *Selection of studies*

Inclusion criteria: We examined the references of published articles, including data on serum PCT for diagnostic sepsis in burned patients. A study was considered eligible for inclusion if it provided both sensitivity and specificity and enough data to construct the 2×2 tables (data corresponding to true positives (TP), true negatives (TN), false positives (FP), and false negatives (FN)). The patients in the included studies principally adults. Exclusion criteria: The studies were excluded which did not afford enough data to construct the 2×2 tables even after contacting the authors. The studies including only pediatric patients were excluded. In order to assure the quality of included studies and exclude the poorly designed or executed studies, the QUADAS (quality assessment of diagnostic accuracy studies) tool [48] was used and the score was at least 10. Selection of articles was conducted separately by four researchers who have diverse educational and professional backgrounds.

2.3. *Data extraction*

Characteristics such as the patients' sample numbers, age, Burned Surface Area, assay method, the cut-off level of PCT, the sensitivity/specificity, and the positive/negative predictive value (PPV/NPV) of PCT for the diagnosis of sepsis in burned patients were extracted. In cases in which major discrepancies between the data reported in the included studies and the data calculated were observed, we contacted the first or last authors of the individual studies via e-mail, requesting clarification regarding the raw data of the patient groups.

2.4. *Quality assessment*

Quality assessment was performed based on the QUADAS (quality assessment of diagnostic accuracy studies) tool [48]. The criteria include 14 items covering several dimensions of study quality: reference independent of index test, same reference standard used, all patients verified by reference standards, short time period between reference and index test, adequate reference standard, selection criteria clearly described, representative spectrum of patients, withdraw explained, unintertable test result reports, clinical data available, blinding for index test, adequate reference test, adequate index description. Each item was assessed by scoring as "low (0 score)", "high (1 score)", or "unclear (−1 score)". The QUADAS score was the sum of the 14 items. The score of each item was discussed by the four authors. The highest score of QUADAS is 15. In order to assure the quality of included studies, all of them should meet at least 70% of the 14 items of QUADAS (QUADAS score of $\geq$10).

2.5. *Statistical analysis*

This meta-analysis was performed with the Meta-Disc 1.4 free software to calculate the pooled sensitivity as TP/(TP + FN), specificity as TN/(TN + FP), positive likelihood ratio (PLR) as (TP/(TP + FN))/(FP/(TN + FP)), negative likelihood ratio (NLR) as (FN/(TP + FN))/(TN/(TN + FP)) and diagnostic odds ratio (DOR) as (TP/FP)/(FN/TN) along with their 95% confidence intervals (CIs).

The summary receiver-operating characteristic (SROC) curve was constructed and the area under the curve (AUC) was then calculated. Analysis of heterogeneity between studies was done using the χ^2 test, which represents the proportion of inter-study variation that can be contributed to heterogeneity rather than to chance. When there was no significant heterogeneity between studies ($P > 0.1$, $I^2 \leq 50\%$), we used fixed-effect meta-analysis. If there was statistical heterogeneity between studies, the meta-analysis was performed using the random-effects model ($P \leq 0.1$, $I^2 > 50\%$). To probe the threshold effect, the Spearman correlation coefficient with Moses' model was calculated. A p-value less than 0.05 indicated significant threshold effect. The Deeks test [49] was performed to detect the publication bias with the Stata 12.1 software, for which a p-value less than 0.1 was suggestive of significant bias.

3. Results

The flow chart of selecting studies was shown as Fig. 1. Nine eligible articles were included. The main characteristics and relative diagnostic data are listed in Tables 1 and 2. The quality assessment was performed strictly based on the QUADAS criteria [48].

3.1. *The accuracy of the PCT test in the diagnosis of sepsis in the burned patients*

We also quantified the effects of heterogeneity using the I^2 test (ranges from 0 to 100%), which represents the proportion of inter-study variation that can be contributed to heterogeneity rather than to chance. I^2 values of 25, 50, and 75% indicate low, moderate, and high degrees of heterogeneity, respectively. The heterogeneity analysis revealed less homogenity for inter-study

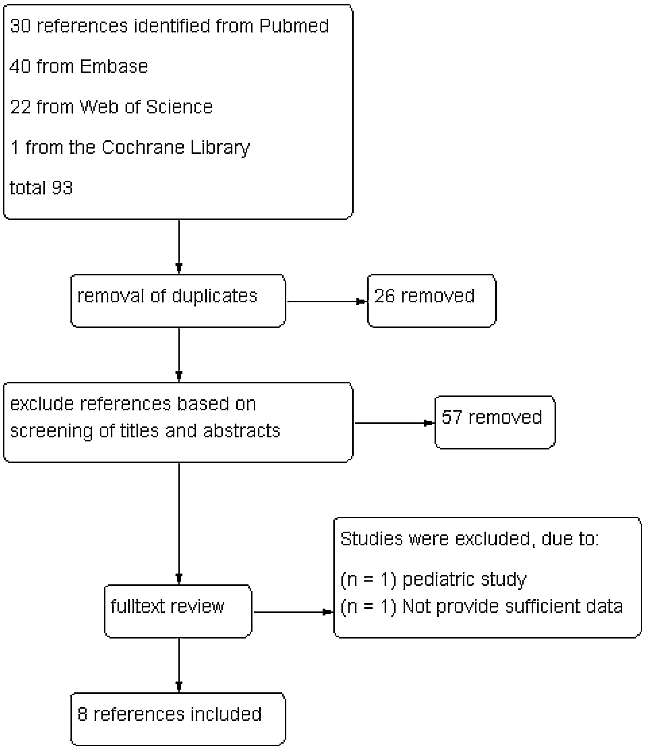

Fig. 1 – Flow chart of study evaluation and inclusion in the meta-analysis of studies.

variation (Chi-square test: sensitivity, $I^2 = 91.1\%$, $P = 0.00$; specificity, $I^2 = 51.1\%$, $P = 0.05$). The meta-analysis was performed using the random-effects model. The sensitivity ranged from 0.11 to 1 (pooled sensitivity: 0.74, 95% CI 0.68–0.79), Whereas specificity varied from 0.76 to 1 (pooled specificity: 0.88, 95% CI 0.84–0.92) (showed in Fig. 2A and B). The pooled positive likelihood ratio is 5.75 [95% CI 3.79, 8.72], the pooled negative likelihood ratio is 0.33 [95% CI 0.15, 0.77], and the pooled diagnostic odds ratio (DOR) is 22.58 [95% CI 8.95, 57.01] (showed in Fig. 3A–C). The area under the receiver operating characteristic (ROC) curve was 0.92 (showed in Fig. 3D).

3.2. *Analysis of threshold effect*

The Spearman's correlation coefficient was 0.41 and the *p* value was 0.32. It shows that no statistical threshold effect, and the probability of heterogeneity caused by different cutoffs was small.

3.3. *Publication bias*

To detect publication bias, we constructed Deeks' funnel plots (the effective sample size funnel plots versus the log diagnostic odds ratio) and performed a Deeks' test (regression test of asymmetry) [49]. The *p* value in the Deeks' test less than 0.1 was suggestive of significant bias. The *p* value in our results is 0.47 (as showed in Fig. 4), indicating publication bias was not identified as a main source of this heterogeneity. However, the number of included studies is small, so the result may be biased.

Table 1 – Main characteristics of studies included in the meta-analysis.

Study and year	Sample size (*n*)	Age (years)	TBSA (%) burned	Cut-off (ng/ml)	Test method
Heimburg, 1998	27	37.3 (18–65)	51 (20–91)	3	ILMA
Bargues, 2007	25	40 ± 14	40 ± 17	0.534	ILMA
Lavrentieva, 2007	43	45.6 ± 20.1	41.4 ± 22	1.5	ILMA
Barati, 2008	60	31.28 ± 17.01*	62.31 ± 20.57*	0.5	ILMA
		30.90 ± 16.78**	57.87 ± 16.89**		
Lavrentieva, 2012	145	48.2 ± 18.3	38.8 ± 18	1.5	ILMA
Kim, 2012	175	45.0 (3–86)	40.0 (1–100)	2	ELFA
Cakır, 2013	37	40 ± 17	36.1 ± 23.4	0.759	ECLIA
Seoane, 2014	34	52.5 ± 17.2	37.6 ± 22.9	1.7	ECLIA

* Patients with sepsis.
** Patients without infection; TBSA: total body surface area; ILMA: immunoluminometric assay, ECLIA: electrochemiluminescence immunoassay, ELFA: enzyme linked fluorescence assay.

Table 2 – Tp, Fp, Fn, Fn, Se, Sp and QUADAS of included studies.

Study and year	Tp	Fp	Fn	Tn	Se (%)	Sp (%)	QUADAS
Heimburg, 1998	2	0	16	9	11	100	10
Bargues, 2007	3	2	4	16	43	89	11
Lavrentieva, 2007	7	3	2	31	82	91.2	13
Barati, 2008	30	3	0	27	100	89.3	11
Lavrentieva, 2012	64	5	9	67	88.3	92.3	14
Kim, 2012	72	15	21	67	77	82	10
Cakır, 2013	13	4	4	16	75.7	78.6	11
Seoane, 2014	4	0	12	18	25	100	10

Tp: true positive; Fp: false positive; Fn: false negative; Tn: true negative; Se: sensitivity; Sp: specificity; and QUADAS: quality assessment of diagnostic accuracy studies.

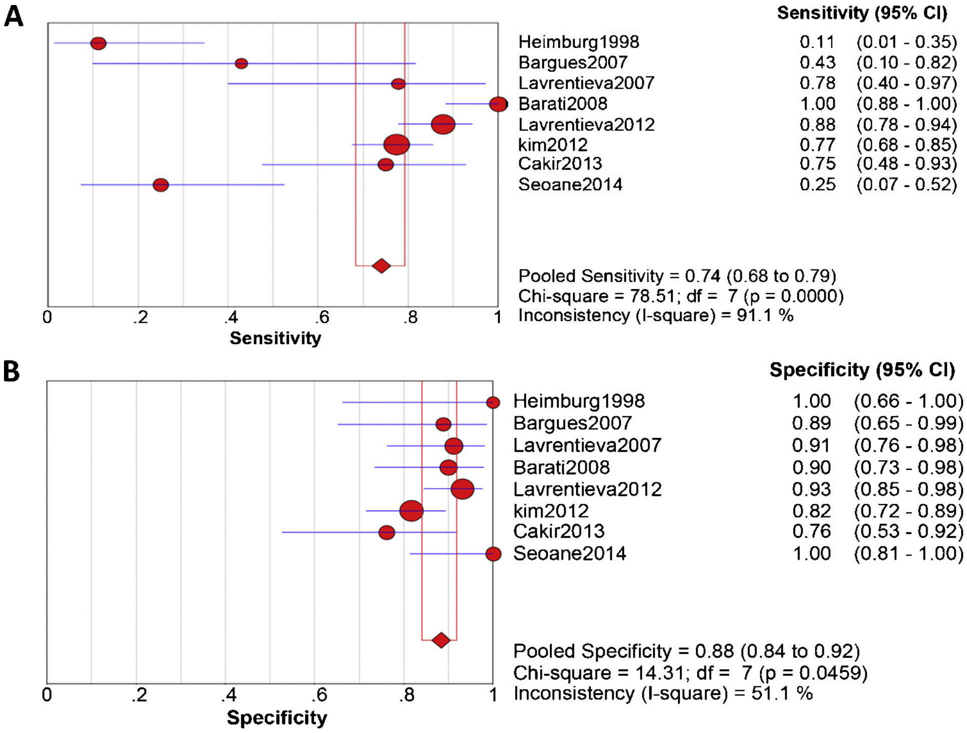

Fig. 2 – Forest plot for pooled sensitivity (A) and specificity (B) of PCT test to diagnose sepsis in burned patients.

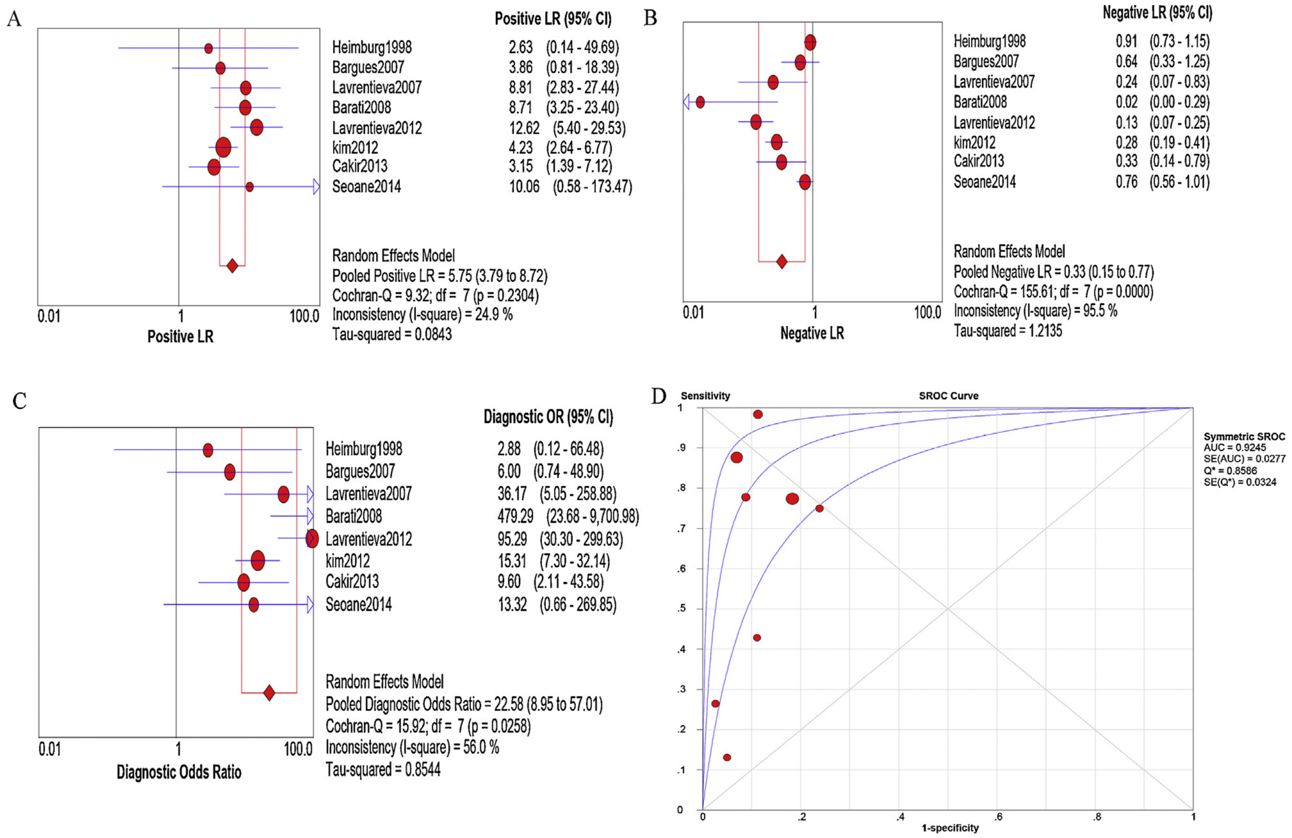

Fig. 3 – Forest plot for pooled positive and negative likelihood ratio ((A) and (B)), diagnostic odds ratio (C) and the SROC (D) of PCT for the diagnosis of sepsis in burned patients.

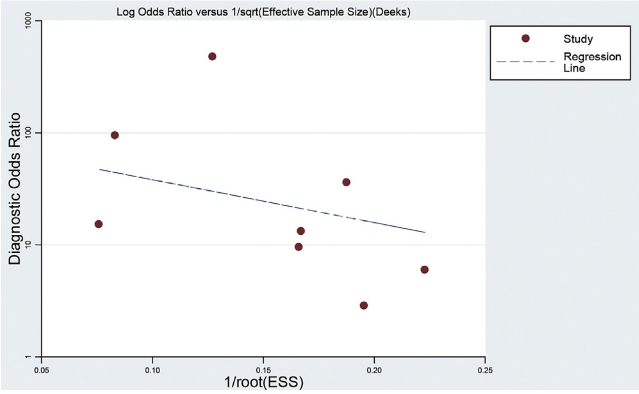

Fig. 4 – Deeks test for the publication bias on the pooled DOR of PCT for the diagnosis of sepsis in burned patients. No significant statistical publication bias was detected in this meta-analysis.

4. Discussion

Sepsis is the most common cause of death in burned patients [4–7]. Delay in diagnosis and treatment often results in rapid progression to circulatory collapse, multiple organ failure, and eventually death [8–10]. Therefore, accurate and timely diagnosis will limit morbidity, reduce costs, and improve patients' outcome [50–52]. However, patients who have sustained a severe burn demonstrate an overwhelming hyper metabolic and hyperinflammatory response [53]. This leads to physiological changes, including persistent tachycardia and tachypnea as well as resetting of the baseline temperature at a higher level [12]. These changes mean that virtually all patients with burns demonstrate signs of SIRS giving them little discriminative value in the diagnosis or prognosis of patients with burns. It is very difficult to diagnose sepsis early because the clinical signs, and the routine laboratory tests are always unreliable [29].

Thus, more effective biomarkers are needed in early diagnosis of sepsis in burned patients. Mann [29] systematically reviewed the effect of PCT in diagnosing sepsis in burn patients in 2011. Since then, some new larger-scale articles have been published. We hence performed a meta-analysis to verify the PCT for diagnosing sepsis in burned patients.

According to our findings, the included studies were published from 1998 to 2014. Some detailed laboratory tests procedures of these techniques could have been modified during this long period. The laboratory test methods for PCT analysis include immunoluminometric assay, electrochemiluminescence immunoassay, and enzyme-linked fluorescence assay. The number of patients in these included studies varied from 25 to 175. The larger-scale studies weigh more than the smaller ones in those pooled data. Most of the included patients were adults. The entire pediatric study was excluded to assure the homogeneity.

Through the meta-analysis, the area under the SROC curve was 0.92, and the pooled sensitivity and specificity were 0.74 and 0.88, respectively. The pooled results of these eight studies analyzed by patients showed that the serum PCT has a moderate level of utility in the diagnosis of sepsis in burned patients. However, the individual studies show varied sensitivities (0.11–1) and specificities (0.76–1). The specificities seems much more stable than the sensitivities. This implies PCT has a higher ability of differential sepsis from non-sepsis but is not sensitive in some situations. It was found that the stability of sensitivities increased when the patients numbers rose (as Lavrentieva's and Kim's studies showed in Fig. 2A).

The pooled positive-likelihood-ratio is 5.75 and the pooled negative-likelihood-ratio is 0.33. A likelihood ratio of greater than 1 indicates the test result is associated with the disease. A likelihood ratio less than 1 indicates that the result is associated with the absence of the disease. In our results, a positive likelihood ratio of 5.75 implies that a person with sepsis is 5.75 times more likely to have a positive test result than is a healthy person. The pooled likelihood-ratio shows that PCT can differentiate sepsis from non-sepsis.

The diagnostic odds ratio (DOR) equals to (TP/FP)/(FN/TN) and can act as an ideal independent indicator which combines the strengths of sensitivity and specificity. The value of a DOR could ranges from 0 to infinity. Higher values indicate better discriminatory test performance. The value of 1 means that the test can not differentiate patients with the disorder or without it [54]. In our study, the pooled diagnostic odds ratio (DOR) was 22.58, indicating a moderate level of overall accuracy for diagnosing sepsis.

The cut-offs of PCT among the included studies vary from 0.534–3 ng/ml. We found that the cut-offs are variable even when using the same test method [40,39]. For example, the cut-offs in Heimburg' (3 ng/ml) and Barati' (0.5 ng/ml) studies varies widely when using the same immunoluminometric assay. The difference may result from the number of patients and burned surface area. The sensitivities were also different between the studies of Bargues' (43%) and Barati' (100%) though the cut-offs are similar (0.534 versus 0.53). The main difference seems also lies in the number of patients and burned surface area. We hence infer the burned surface area may have some potential correlation to the PCT level and the number of patients may affect the reliability of results.

Moreover, PCT can act as not only a diagnostic tool for sepsis but also a prognostic index for the burn patients. Kim [55] reported that the burn patients' procalcitonin levels at the time of their admission to the hospital could serve as a prognostic marker. It could be very useful to verify whether the PCT have a prognostic effect in burned patients. However, the data in these relative studies are not sufficient to perform a meta-analysis on the prognostic effect of PCT, which would be a very meaningful topic for future studies.

As our results show, PCT may not be the most ideal biomarker for early diagnosis of sepsis in burned patients. To our knowledge, an absolutely ideal biomarker may do not exist because sepsis is a complex pathophysiological process and difficult to be described by a single biomarker. In general, we should give a relative affirmative view of PCT. It could be a useful and one of the most promising sepsis biomarkers in burned patients.

There were some limitations in our study, though we have tried our best to minimize the biases by using a compound thorough search, making an ideal criteria for inclusion and exclusion, consulting the statistician and experts in the

department of Clinical Laboratory, and making group discussions when needed.

First, the sample size in the included articles is relatively small. Only eight studies were enrolled in the meta-analysis. We tried to include all eligible published articles by a thorough search of databases and remove articles which can't meet the criteria. Sachse C [47] reported on procalcitonin as a marker for the diagnosis of severe infection after thermal injury. However, the study [47] can't provide sufficient data to construct the 2 $\times$ 2 tables. It was finally excluded after review of the full-text and connected with the authors.

In order to assure the quality of included studies, we assessed the studies with QUADAS score. All included studies should meet at least 70% of the 14 items of QUADAS (QUADAS score of $\geq$10). However, the numbers of burn patients in most included studies [20,44,40,39,42,41] were too small ($\leq$60) to obtain conclusion although the QUDAS results were acceptable. Only two studies [43,45] have relative larger patient numbers (>60) and have similar results, including cut-offs, sensitivities and specificities. To avoid the bias and get better homogeneity from patients' ages, we exclude the studies which only include the pediatric population [46]. To our knowledge, the physiological characteristics and body response to burn in children are totally different from those of the adult [56]. We finally can't perform a subgroup analysis. For pediatric burn patients, sepsis is the leading cause of death [57]. So, further researches on it are needed in the future.

Second, threshold effect was one of the common causes of heterogeneity among diagnostic accuracy studies. The cut-offs or thresholds can define a positive or negative test result. The cut-offs may have some correlations with the burned surface area which affect the release of PCT. Although no statistical significant threshold effect was found, the diverse cut-off values (ranges from 0.5 to 3 ng/ml) potentially lead to the heterogeneity among the studies. Three kinds of assays were used in the eight included studies. However, a major advantage in comparing these studies originates from the availability of standard antibodies against procalcitonin. As reported in each paper, procalcitonin was measured in all the studies by using the same antibody (Brahms) and this is an advantage for standardization of the results. In addition, such studies were performed on a small number of patients, but on a great number of samples and this means robust results.

To reduce the affect of threshold effect, evaluating individual PCT trends will be more important than absolute values.

Third, the time point of blood samples harvesting are also not uniform. The PCT levels at the onset of sepsis were different from those at a septic shock peak period or recover period. To diagnose the sepsis earlier, the onset PCT levels seem more meaningful than other time points. However, the PCT may not rise as quickly as the clinical manifestation changes. Keep tracking the changing tendency of PCT levels could be a meaningful method to know the development of sepsis in burned patients.

Fourth, though there are guidelines for the definition of sepsis [25,58] and the concept of Systemic Inflammatory Response Syndrome (SIRS), it has been criticized for being too sensitive and nonspecific [25]. The definition criteria of sepsis for burn patients are not uniform [25,59,60]. This has led to the American Burn Association producing specific consensus guidelines for the definition of infection and sepsis in patients with burns [12]. Experts [61,62] from China also revised the diagnostic criteria of sepsis in burned patients. However, there was still not an ideal definition of sepsis with high sensitivity and specificity for burned patients. So, the main problem in the field is the lack of a uniform definition of sepsis and the difficulty to diagnose sepsis. The results hence could be biased by the lack of a gold standard for the definition of sepsis. Unless a uniform definition of sepsis is available, these limitations will continue to be inherent in the research in this field.

Finally, we only searched the published articles in English. Studies with positive results are more easily to be published, which would affect the estimation of the pooled diagnostic accuracy. The presence of a potential publication bias may hence be existed for the lack of information on the presence of extensive research studies or abstracts unpublished and the studies published in other languages.

In all, although we can get the information that the PCT could be a useful sepsis biomarker in burned patients from the meta-analysis, the results should be interpreted with caution, due to the substantial heterogeneity among study designs. Procalcitonin kinetics (serial procalcitonin testing) could help the early diagnosis of sepsis and reduce the unnecessary use of antibiotics or selective pressure for multiresistant pathogens. Further multi-center larger-scale prospective studies should be done with uniform laboratory's methods and cutoff values to limit the bias. In addition, more studies on PCT in diagnosing sepsis in the pediatric populations are needed.

5. Conclusion

The findings indicate that serum PCT could be a useful sepsis biomarker in burned patients. However, it must be used cautiously and cannot act as the single definitive test for sepsis diagnosis. The diagnosis should be based on integrating ambulatory monitoring biological markers with clinical parameters such as relative medical history, careful physical examination, and microbiological assessment. The trace of PCT changes during disease would be more important than absolute values.

Conflict of interest statement

We declare that we have no conflicts of interest.

Acknowledgments

The project was supported by the Health Department of the Zhejiang Province, (No. 201345919), China and the Teaching Department of the Zhejiang Province (No. Y201330073). We thank the statistician Zexin Chen and Xiaofei Zhang in the Department of Epidemiology and Dr. Hai-en Cheng in the department of Clinical Laboratory in the Second Affiliated Hospital, School of Medicine, Zhejiang University. Haitao Ren and Yuan Li contributed equally to this work and should be considered co-first authors.

REFERENCES

[1] Hotchkiss RS, Monneret G, Payen D. Sepsis-induced immunosuppression: from cellular dysfunctions to immunotherapy. Nat. Rev. Immunol. 2013;13(12): 862–874.

[2] Vincent J-L, Opal SM, Marshall JC, Tracey KJ. Sepsis definitions: time for change. Lancet 2013;381(9868): 774–775.

[3] Fitzwater J, Purdue GF, Hunt JL, O'Keefe GE. The risk factors and time course of sepsis and organ dysfunction after burn trauma. J. Trauma 2003;54(5):959–66.

[4] D'Avignon LC, Hogan BK, Murray CK, Loo FL, Hospenthal DR, Cancio LC, et al. Contribution of bacterial and viral infections to attributable mortality in patients with severe burns: an autopsy series. Burns 2010;36(6):773–9.

[5] Sharma BR, Harish D, Singh VP, Bangar S. Septicemia as a cause of death in burns: an autopsy study. Burns 2006;32(5):545–9.

[6] Prasad J, Feller I, Thomson P. A ten-year review of *Candida sepsis* and mortality in burn patients. Surgery 1987;101(2):213.

[7] Chipp E, Milner CS, Blackburn AV. Sepsis in burns: a review of current practice and future therapies. Ann Plast Surg 2010;65(2):228–36.

[8] Butt MA, Coulson AK, Hull JH, Ho TB. Bloodstream infection: early treatment strategies in sepsis. BMJ 2008;336(7643):521.

[9] Warren HS. Strategies for the treatment of sepsis. N Engl J Med 1997;336(13):952–3.

[10] Riedemann NC, Guo RF, Ward PA. Novel strategies for the treatment of sepsis. Nat Med 2003;9(5):517–24.

[11] Levy MM, Fink MP, Marshall JC, Abraham E, Angus D, Cook D, et al. 2001 SCCM/ESICM/ACCP/ATS/SIS international sepsis definitions conference. Crit Care Med 2003;31(4):1250–6.

[12] Greenhalgh DG, Saffle JR, Holmes JHt, Gamelli RL, Palmieri TL, Horton JW, et al. American Burn Association consensus conference to define sepsis and infection in burns. J Burn Care Res 2007;28(6):776–90.

[13] Chiesa C, Panero A, Osborn JF, Simonetti AF, Pacifico L. Diagnosis of neonatal sepsis: a clinical and laboratory challenge. Clin Chem 2004;50(2):279–87.

[14] Schelonka RL, Chai MK, Yoder BA, Hensley D, Brockett RM, Ascher DP. Volume of blood required to detect common neonatal pathogens. J Pediatr 1996;129(2):275–8.

[15] Finnerty CC, Herndon DN, Przkora R, Pereira CT, Oliveira HM, Queiroz DM, et al. Cytokine expression profile over time in severely burned pediatric patients. Shock (Augusta, GA) 2006;26(1):13–9.

[16] Murray CK, Hoffmaster RM, Schmit DR, Hospenthal DR, Ward JA, Cancio LC, et al. Evaluation of white blood cell count, neutrophil percentage, and elevated temperature as predictors of bloodstream infection in burn patients. Arch Surg (Chicago, IL: 1960) 2007;142(7):639–42.

[17] Jones AE, Fiechtl JF, Brown MD, Ballew JJ, Kline JA. Procalcitonin test in the diagnosis of bacteremia: a meta-analysis. Ann Emerg Med 2007;50(1):34–41.

[18] Simon L, Gauvin F, Amre DK, Saint-Louis P, Lacroix J. Serum procalcitonin and C-reactive protein levels as markers of bacterial infection: a systematic review and meta-analysis. Clin Infect Dis 2004;39(2):206–17 (An official publication of the Infectious Diseases Society of America).

[19] Uzzan B, Cohen R, Nicolas P, Cucherat M, Perret GY. Procalcitonin as a diagnostic test for sepsis in critically ill adults and after surgery or trauma: a systematic review and meta-analysis. Crit Care Med 2006;34(7):1996–2003.

[20] Seoane L, Pertega S, Galeiras R, Astola I, Bouza T. Procalcitonin in the burn unit and the diagnosis of infection. Burns 2014;40(2):223–9.

[21] Assicot M, Bohuon C, Gendrel D, Raymond J, Carsin H, Guilbaud J. High serum procalcitonin concentrations in patients with sepsis and infection. Lancet 1993;341(8844):515–8.

[22] Brunkhorst F, Wegscheider K, Forycki Z, Brunkhorst R. Procalcitonin for early diagnosis and differentiation of SIRS, sepsis, severe sepsis, and septic shock. Intensive Care Med 2000;26(2):S148–52.

[23] Aouifi A, Piriou V, Bastien O, Blanc P, Bouvier H, Evans R, et al. Usefulness of procalcitonin for diagnosis of infection in cardiac surgical patients. Crit Care Med 2000;28(9):3171–6.

[24] Al-Nawas B, Krammer I, Shah P. Procalcitonin in diagnosis of severe infections. Eur J Med Res 1996;1(7):331–3.

[25] Levy MM, Fink MP, Marshall JC, Abraham E, Angus D, Cook D, et al. 2001 SCCM/ESICM/ACCP/ATS/SIS international sepsis definitions conference. Intensive Care Med 2003;29(4):530–8.

[26] Wacker C, Prkno A, Brunkhorst FM, Schlattmann P. Procalcitonin as a diagnostic marker for sepsis: a systematic review and meta-analysis. Lancet Infect Dis 2013;13(5):426–35.

[27] Zhao D, Zhou J, Haraguchi G, Arai H, Mitaka C. Procalcitonin for the differential diagnosis of infectious and non-infectious systemic inflammatory response syndrome after cardiac surgery. J Intensive Care 2014;2(1):35.

[28] Schneider H-G, Lam QT. Procalcitonin for the clinical laboratory: a review. Pathology 2007;39(4):383–90.

[29] Mann EA, Wood GL, Wade CE. Use of procalcitonin for the detection of sepsis in the critically ill burn patient: a systematic review of the literature. Burns 2011;37(4):549–58.

[30] Uzzan B, Cohen R, Nicolas P, Cucherat M, Perret G-Y. Procalcitonin as a diagnostic test for sepsis in critically ill adults and after surgery or trauma: a systematic review and meta-analysis. Crit Care Med 2006;34(7):1996–2003.

[31] Vincent J-L. Procalcitonin: THE marker of sepsis? Crit Care Med 2000;28(4):1226–8.

[32] Lapillonne A, Basson E, Monneret G, Bienvenu J, Salle BL. Lack of specificity of procalcitonin for sepsis diagnosis in premature infants. Lancet 1998;351(9110):1211–2.

[33] Ruokonen E, Ilkka L, Niskanen M, Takala J. Procalcitonin and neopterin as indicators of infection in critically ill patients. Acta Anaesthesiol Scand 2002;46(4):398–404.

[34] Ugarte H, Silva E, Mercan D, De Mendonca A, Vincent J-L. Procalcitonin used as a marker of infection in the intensive care unit. Critical Care Med 1999;27(3):498–504.

[35] Harbarth S, Holeckova K, Froidevaux CL, Pittet D, Ricou B, Grau GE, et al. Diagnostic value of procalcitonin, interleukin-6, and interleukin-8 in critically ill patients admitted with suspected sepsis. Am J Respir Crit Care Med 2001;164(3):396–402.

[36] Müller B, Becker KL, Schächinger H, Rickenbacher PR, Huber PR, Zimmerli W, et al. Calcitonin precursors are reliable markers of sepsis in a medical intensive care unit. Critical Care Med 2000;28(4):977–83.

[37] Christ-Crain M, Jaccard-Stolz D, Tamm M, Müller B. Procalcitonin-guided antibiotic treatment in heart failure. Lancet 2004;363(9420):1555–6.

[38] Tang BM, Eslick GD, Craig JC, McLean AS. Accuracy of procalcitonin for sepsis diagnosis in critically ill patients: systematic review and meta-analysis. Lancet Infect Dis 2007;7(3):210–7.

[39] Barati M, Alinejad F, Bahar MA, Tabrisi MS, Shamshiri AR, Bodouhi NO, et al. Comparison of WBC, ESR, CRP and PCT serum levels in septic and non-septic burn cases. Burns 2008;34(6):770–4.

[40] Bargues L, Chancerelle Y, Catineau J, Jault P, Carsin H. Evaluation of serum procalcitonin concentration in the ICU following severe burn. Burns 2007;33(7):860–4.
[41] Lavrentieva A, Kontakiotis T, Lazaridis L, Tsotsolis N, Koumis J, Kyriazis G, et al. Inflammatory markers in patients with severe burn injury. What is the best indicator of sepsis? Burns 2007;33(2):189–94.
[42] von Heimburg D, Stieghorst W, Khorram-Sefat R, Pallua N. Procalcitonin—a sepsis parameter in severe burn injuries. Burns 1998;24(8):745–50.
[43] Lavrentieva A, Papadopoulou S, Kioumis J, Kaimakamis E, Bitzani M. PCT as a diagnostic and prognostic tool in burn patients. Whether time course has a role in monitoring sepsis treatment. Burns 2012;38(3):356–63.
[44] Çakır Madenci Ö, Yakupoğlu S, Benzonana N, Yücel N, Akbaba D, Orçun Kaptanağası A. Evaluation of soluble CD14 subtype (presepsin) in burn sepsis. Burns 2013.
[45] Kim HS, Yang HT, Hur J, Chun W, Ju Y-S, Shin S-H, et al. Procalcitonin levels within 48 h after burn injury as a prognostic factor. Ann Clin Lab Sci 2012;42(1):57–64.
[46] Neely AN, Fowler LA, Kagan RJ, Warden GD. Procalcitonin in pediatric burn patients: an early indicator of sepsis? J Burn Care Res 2004;25(1):76–80.
[47] Sachse C, Machens HG, Felmerer G, Berger A, Henkel E. Procalcitonin as a marker for the early diagnosis of severe infection after thermal injury. J Burn Care Rehabil 1999;20(5):354–60.
[48] Whiting P, Rutjes AW, Reitsma JB, Bossuyt PM, Kleijnen J. The development of QUADAS: a tool for the quality assessment of studies of diagnostic accuracy included in systematic reviews. BMC Med Res Methodol 2003;3:25.
[49] Deeks JJ, Macaskill P, Irwig L. The performance of tests of publication bias and other sample size effects in systematic reviews of diagnostic test accuracy was assessed. J Clin Epidemiol 2005;58(9):882–93.
[50] Pittet D, Rangel-Frausto S, Li N, Tarara D, Costigan M, Rempe L, et al. Systemic inflammatory response syndrome, sepsis, severe sepsis and septic shock: incidence, morbidities and outcomes in surgical ICU patients. Intensive Care Med 1995;21(4):302–9.
[51] Kollef MH, Sherman G, Ward S, Fraser VJ. Inadequate antimicrobial treatment of infections: a risk factor for hospital mortality among critically ill patients. Chest 1999;115(2):462–74.
[52] Bochud PY, Glauser MP, Calandra T. Antibiotics in sepsis. Intensive Care Med 2001;27(Suppl 1):S33–48.
[53] Atiyeh BS, Gunn SW, Dibo SA. Metabolic implications of severe burn injuries and their management: a systematic review of the literature. World J Surg 2008;32(8):1857–69.
[54] Glas AS, Lijmer JG, Prins MH, Bonsel GJ, Bossuyt PM. The diagnostic odds ratio: a single indicator of test performance. J Clin Epidemiol 2003;56(11):1129–35.
[55] Kim HS, Yang HT, Hur J, Chun W, Ju YS, Shin SH, et al. Procalcitonin levels within 48 h after burn injury as a prognostic factor. Ann Clin Lab Sci 2012;42(1):57–64.
[56] Peng YZ. Clinical characteristics and diagnosis of sepsis in pediatric burn patients. Zhonghua shaoshang zazhi Chin J Burns2013;29(1):1–3.
[57] Williams FN, Herndon DN, Hawkins HK, Lee JO, Cox RA, Kulp GA, et al. The leading causes of death after burn injury in a single pediatric burn center. Critical Care (London, England) 2009;13(6):R183.
[58] American College of Chest Physicians/Society of Critical Care Medicine Consensus Conference: definitions for sepsis and organ failure and guidelines for the use of innovative therapies in sepsis. Crit Care Med 1992;20(6):864–74.
[59] Greenhalgi D, Saffle J, Holmes J. American Burn Association consensus conference to define sepsis and infections in burns. J Burn Care Res 2007;28:776–90.
[60] Bone RC, Balk RA, Cerra FB, Dellinger RP, Fein AM, Knaus WA, et al. Definitions for sepsis and organ failure and guidelines for the use of innovative therapies in sepsis. The ACCP/SCCM Consensus Conference Committee. American College of Chest Physicians/Society of Critical Care Medicine. CHEST J 1992;101(6):1644–55.
[61] Yizhi P, Jing C, Zhiqiang Y, Xiaolu L, Gaoxing L, Jun W. Diagnostic criteria and treatment protocol for post-burn sepsis. Crit. Care 2013;17(1):406.
[62] Peng Y, Yuan Z. Standardized definitions and diagnostic criteria for infection in burn patients. Zhonghua shaoshang zazhi Chin J Burns2007;23(6):404.
