# Supplementary material for: The Use of Procalcitonin (PCT) for Diagnosis of Sepsis in Burn Patients: A Meta-Analysis
Source: PLoS One. 2016 Dec 22;11(12):e0168475. doi: 10.1371/journal.pone.0168475 (PMC5179235; doi:10.1371/journal.pone.0168475)
Supplement: S3 File — Mann. (PDF) [file pone.0168475.s003.pdf]

available at www.sciencedirect.com

journal homepage: www.elsevier.com/locate/burns

Review

# Use of procalcitonin for the detection of sepsis in the critically ill burn patient: A systematic review of the literature☆

Elizabeth A. Mann[a,c,*], Geri L. Wood[a,b], Charles E. Wade[c]

[a] University of Texas Health Sciences Center, Houston, TX - School of Nursing, United States
[b] MD Anderson Cancer Center, Houston, TX, United States
[c] US Army Institute of Surgical Research, San Antonio, TX, United States

ARTICLE INFO

*Article history:*
Accepted 22 April 2010

*Keywords:*
Procalcitonin
Sepsis
Intensive care
Burn
Diagnosis

ABSTRACT

The purpose of this systematic review was to assess the evidence for use of routine procalcitonin testing to diagnose the presence of sepsis in the burn patient. The electronic databases MEDLINE, Cochrane, CINAHL, ProQuest, and SCOPUS were searched for relevant studies using the MeSH terms burn, infection, procalcitonin, and meta-analysis. The focus of the review was the adult burn population, but other relevant studies of critically ill patients were included as data specific to the patient with burns are limited. Studies were compiled in tabular form and critically appraised for quality and level of evidence. Four meta-analyses, one review of the literature, one randomized controlled trial, nine prospective observational, and three retrospective studies were retrieved. Six of these studies were specific to the burn population, with one specific to burned children. Only one meta-analysis, one adult burn and one pediatric burn study reported no benefit of procalcitonin testing to improve diagnosis of sepsis or differentiate sepsis from non-infectious systemic inflammatory response. The collective findings of the included studies demonstrated benefit of incorporating procalcitonin assay into clinical sepsis determination. Evaluation of the burn specific studies is limited by the use of guidelines to define sepsis and inconsistent results from the burn studies. Utility of the procalcitonin assay is limited due to the lack of availability of rapid, inexpensive tests. However, it appears procalcitonin assay is a safe and beneficial addition to the clinical diagnosis of sepsis in the burn intensive care unit.

Published by Elsevier Ltd and ISBI.

## Contents

1. Introduction . . . . . 550
2. Methods . . . . . 550
3. Results . . . . . 551
   3.1. Meta-analyses and review of the literature . . . . . 551
   3.2. Clinical trials . . . . . 552

☆ The opinions or assertions contained herein are the private views of the authors and are not to be construed as official or as reflecting the views of the Department of the Army or the Department of Defense.

* *Corresponding author at*: 335 Pershing Ave, San Antonio, TX 78209, United States. Tel.: +1 210 467 9246.
E-mail address: Elizabeth.Mann@us.army.mil (E.A. Mann).

0305-4179/$36.00. Published by Elsevier Ltd and ISBI.
doi:10.1016/j.burns.2010.04.013

3.3. Burn specific clinical trials . . . . . . . . . . 554
4. Discussion . . . . . . . . . . 556
Acknowledgements . . . . . . . . . . 557
References . . . . . . . . . . 557

## 1. Introduction

Severe burn frequently results in multiple organ dysfunction and sepsis [1]. The cause of death in 28–65% of fatal burn cases has been attributed to sepsis [2,3]. Yet due to chronic baseline inflammatory response [4] and immune dysregulation [5] the traditional markers of acute infection are difficult to identify in the burn patient. Consensus definitions for sepsis in the critically ill population couple criteria for systemic inflammatory response syndrome (SIRS) with the documented presence of infection [6]. However, the SIRS criteria of more than one of the following clinical findings of temperature >38 °C or <36 °C; heart rate (HR) >90 beats/min; respiratory rate (RR) > 20/min or $PaCO_2$ <32 mmHg; or white blood cell count (WBC) >12,000 or <4,000 cells/μl are the norm for the hypermetabolic burn patient [7]. A consensus panel for the American Burn Association has developed specific guidelines for the diagnosis of sepsis in the burn patient that include higher thresholds for temperature (>39 °C or <36.5 °C), HR (>110 beats/min) and RR (>25/min) in addition to presence of thrombocytopenia (platelet count < 100,000/mcl), and indications of insulin resistance or feeding intolerance [7]. In addition to these clinical indicators, documented presence of infection or clinical response to antimicrobials is required. Because these guidelines are based on consensus and not founded in prospective clinical studies, more precise methods of detecting sepsis in this vulnerable population are necessary. Evidence of an increased risk of mortality in the burn patient infected with the ubiquitous pathogen *Pseudomonas aeruginosa* is suggested if appropriate antibiotic therapy is delayed for only 2 days [8].

Detection of sepsis would be expedited if a simple, inexpensive test could be performed routinely, with a high degree of accuracy in correctly differentiating sepsis from SIRS. Such an assay should improve the ability to identify severe infection, guide treatment and reduce the duration of antibiotic exposure. Emergence of a test that meets these criteria is the assay of the procalcitonin (PCT) molecule, a precursor of calcitonin, produced in both thyroidal and extrathyroidal tissues, including adipose tissue [9]. Release of PCT occurs to varying degrees in response to bacterial infection, fungal infection, trauma, surgery and other types of conditions. The greatest elevations of serum PCT occur in the presence of bacterial infection and multi-organ failure resulting from trauma [10], and no change is found due to viral infection [9]. Compared to other sepsis markers used clinically such as tumor necrosis factor-alpha (TNF-α), interleukin-6 (IL-6) or C-reactive protein (CRP) the reactive pattern of PCT has an onset within 4 h of response to infection or injury, peaks at 6 h with a plateau of 8–24 h, then returns to baseline in 2–3 days. This is compared to a 90 min onset for TNF-α with return to baseline in 6 h; a 3-h onset for CRP with return to baseline in 8 h; and a 12–24 h onset for CRP with a 20–72-h plateau and 3–7-day return to baseline [11]. The relatively early rise of PCT with a long plateau of up to 24 h after response to sepsis makes this marker ideal for routine daily measurement; a sudden rise in PCT level is an indicator of sepsis onset [9]. The normal serum value of PCT in a healthy individual without inflammation is less than 0.05 ng/mL [9]. PCT levels associated with local infection, possible systemic infection, sepsis, or severe sepsis are: <0.5 ng/mL, 0.5–2 ng/mL, 2–10 ng/mL, and >10 ng/mL respectively [12].

Numerous clinical trials and meta-analyses of ability to detect sepsis in acutely and critically ill populations using PCT assay have produced promising results [13–15]. Multiple studies specifically in the burn population have been performed [16–19]. While European and Asian countries have been the leaders in this new technology; widespread availability or use of the PCT assay in the United States is lacking. A systematic review of the literature was conducted to identify evidence supporting use of the procalcitonin diagnostic test to detect sepsis in the critically ill burn patient.

## 2. Methods

To identify relevant research regarding the usefulness of the procalcitonin test in the early diagnosis of sepsis in the burn patient a systematic review of the literature was performed. MEDLINE, Cochrane Database, CINAHL, ProQuest, and SCOPUS electronic databases were searched in November 2009. Combinations of the MeSH terms burn, procalcitonin, and meta-analysis were searched; reference lists for relevant articles were reviewed for additional pertinent articles. The search was limited to studies of human subjects, clinical trials, and English language. No limits for date were applied to search using burn and procalcitonin or meta-analysis and procalcitonin. Date limits of 2004–2009 were applied to the search of procalcitonin and infection as previously published articles were included in one or more meta-analyses or systematic reviews [13–15,20,21]. Studies considered for inclusion were performed with adult subjects, with an emphasis on burn injury but included other critically ill populations with the diagnosis of sepsis. Exclusion criteria included studies with the predominate focus on prediction of outcome, use of procalcitonin test to guide antibiotic therapy, neonatal subjects or animal studies.

The level of evidence for each study was determined using the American Association of Critical-Care Nurses Evidence leveling system [22]. Meta-analysis is considered Level A; well designed randomized controlled trials with consistent results - Level B; and systematic reviews, descriptive studies or controlled trials with inconsistent results - Level C evidence. Each study was also evaluated using the U.S. Preventive Task Force Quality Rating Criteria for diagnostic accuracy studies [23] resulting in ratings of good, fair or poor based on rating

criteria. The criteria included relevance of screening test, use of a credible reference standard, interpretation of reference standard independent of screening test, in determinant results handled in a reasonable manner, broad spectrum of patients included with adequate sample size, and administration of a reliable screening test.

## 3. Results

A total of 19 articles were included in this review; the systematic process of selection is described in Fig. 1(a–c). Four meta-analyses [13–15,21] and 1 review of the literature [20] (Table 1), were retrieved. Studies conducted after 2004 and not included in the meta-analyses or systematic review included 1 randomized controlled trial (RCT) [24], 10 prospective observational studies [16–19,25–30], and 3 retrospective reviews [31–33] (Table 2). Of these, 5 prospective [16–19,28] and one retrospective study [33] were burn patient specific (Table 3).

### 3.1. *Meta-analyses and review of the literature*

Populations included in the meta-analyses included emergency department (ED) patients [13,21], surgical and medical inpatients [14], and surgical and trauma patients [15]; febrile neutropenic patients were the focus of the review of literature [20]. Collectively these studies evaluated a range of 12–33 individual clinical trials reported from 1996 to 2007, comprised of a range of 1222–2335 subjects, with a subset of 486–603 pediatric subjects included in 3 of the systematic reviews. Significant overlap occurred among these meta-analyses and review of the literature as many of the same studies were included in multiple reviews. Only one meta-analysis determined the PCT assay to fail to distinguish sepsis from SIRS among a mixed sample from the ED, ICU and general inpatient units [21]; the other meta-analyses determined a moderate [13] ability of PCT assay to identify sepsis in an ED population, and superiority of PCT over CRP to identify bacterial infection or sepsis in the inpatient setting [14,15]. The conclusion of the literature review of PCT value in predicting sepsis in the neutropenic population determined the ability of the assay to discriminate infectious etiology in this subset of patients [20].

Quality of the selected meta-analyses appears to be robust. Each study described a comprehensive search strategy and provided a flow diagram or grading criteria for included studies, statistical procedures were conducted appropriately although Tang et al. [21] arrived at contradictory conclusion of PCT performance compared to the other meta-analyses [13–15]. The review of literature [20] did not include statistical analysis but a rigorous search strategy resulted in inclusion of over 30 clinical studies of febrile neutropenic patients, a population akin to the severely burned patient and thus the study was included in this analysis. Level of evidence for 3 of the meta-analyses [13–15] was "A" (results from a meta-analysis that consistently support a specific action), and "C" for the meta-analysis conducted by Tang et al. [21] (systematic reviews or meta-analyses with inconsistent results) [22].

Findings of the meta-analyses resulted in differing conclusions. Uzzan et al. [15] report an odds ratio (OR) of 15.7 (95%

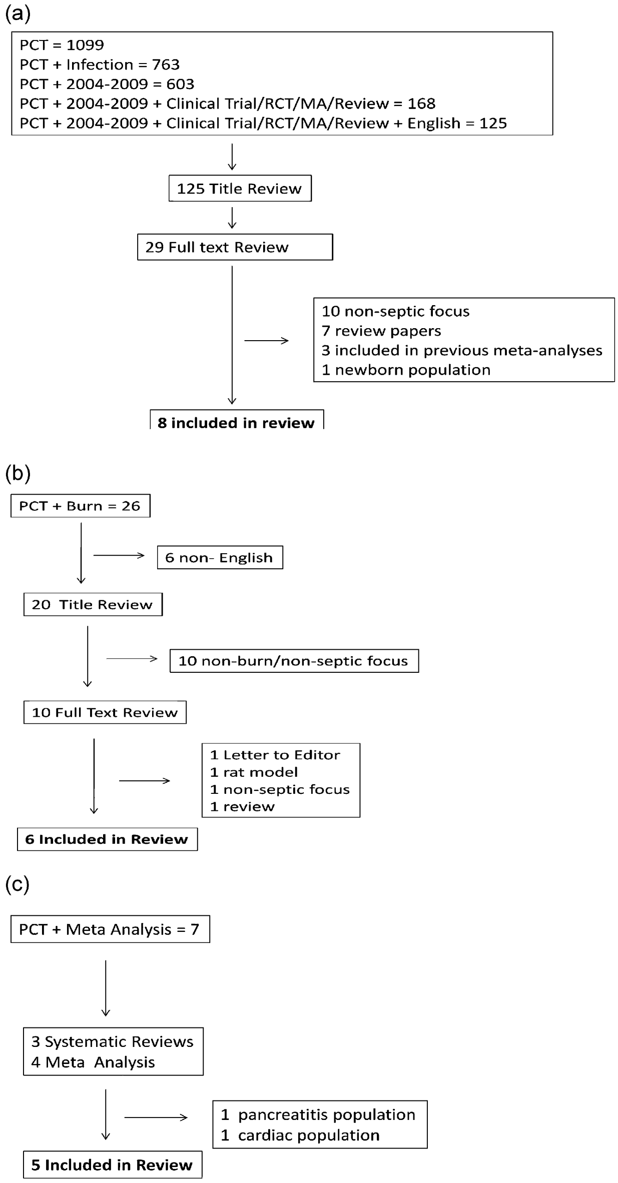


**Fig. 1 – (a–c) Flow diagrams for included studies and search criteria.**

CI, 9.1–27.1, $p < 0.0001$) for PCT test when infection was compared with non-septic SIRS. The diagnostic OR of the PCT assay performance to diagnose sepsis reported by Jones et al. [13] was 9.86 (95% CI, 5.72–17.02). These findings are contrasted to those of Tang et al. [21] where an OR of 7.79 (95% CI, 5.86–10.35) was calculated for the diagnostic ability of PCT test to accurately discriminate between sepsis and non-septic SIRS. Simon et al. [14] reported a pooled sensitivity and specificity for PCT assay of 88% and 81% respectively, compared to assessment by Tang et al. [21] of 71% and 71% with area under the receiver operating curve (AUC) of 0.78; this value contrasts with the AUC reported by Jones et al. [13] of 0.84. The findings of Sakr et al. [20] for the diagnostic ability of the PCT assay to detect sepsis in the febrile neutropenic population determine a cut-off value of PCT >2 ng/mL associated with sepsis and septic shock and values between 1.0 and 2.0 ng/mL to suggest

**Table 1 – Characteristics of included meta-analyses and systematic reviews.**

| Study | Study design | Purpose | Sample | PCT assay | Conclusions | Overall findings |
|---|---|---|---|---|---|---|
| Jones et al. [13] | Meta-analysis | Diagnostic accuracy of PCT with blood culture as reference for bacteremia in ED | 17 studies: 1999–2004; ED population or new admit; Pediatric (7) $n = 603$; adult (10) $n = 1222$; mixed $n = 183$ | NR | Moderate ability of PCT to identify sepsis in ambulatory population in ED | (+) moderate |
| Tang et al. [21] | Meta-analysis | Estimate diagnostic accuracy of PCT in sepsis diagnosis for critically ill patients | 18 studies: 1996–2005; $n = 2097$ (ICU $n = 1452$, ED $n = 440$, ward $n = 205$), all adults | LUMItest | PCT does not accurately distinguish sepsis from SIRS in critically ill adults | (−) |
| Uzzan et al. [15] | Meta-analysis | Accuracy of PCT as diagnostic test for sepsis in ICU after surgery or trauma compared with CRP | 33 studies: 1999–2004; Surgery/trauma, sepsis: $n = 1825$ | NR | PCT superior to CRP, good biological marker for sepsis | (+) |
| Simon et al. [14] | Meta-analysis | Evaluate accuracy of PCT and CRP for diagnosis of bacteremic infection | 12 studies: 1999–2001; Hospitalized pts: $n = 1361$ (9 adult $n = 733$, 3 pediatric $n = 678$) | LUMItest | Accuracy of PCT higher than CRP in differentiation of bacteremic infection from non-infectious inflammation | (+) |
| Sakr et al. [20] | Literature review | Value of PCT for predicting infection in neutropenic patients | 30 studies: 1996–2007; Febrile neutropenia; $n = 2335$ (adult $n = 1499$, pediatric $n = 486$, mixed $n = 350$) | LUMItest $n = 29$; PCT-Q test $n = 1$ | PCT able to discriminate infectious etiology in neutropenic patients | (+) |

CRP: C-reactive protein; ED: Emergency Department; ICU: intensive care unit; NR: not reported; PCT: procalcitonin.

infection due to bacteremia or fungal infection. Overall study results demonstrated the PCT assay to be a useful diagnostic test for sepsis, with only one meta-analysis not recommending widespread use of the test [21].

### 3.2. Clinical trials

Of the clinical trials conducted from 2006 to 2009 without a burn patient focus was: 1 RCT [24], 5 prospective clinical trials [25–27,29,30] and 2 retrospective chart reviews [31,32]. All studies were conducted outside of the United States, 7 in European countries [24,25,27,30–32] and 2 in Asia [26,29]. The populations in the prospective trials ranged from Emergency Department ($n = 80$) [25], inpatients with SIRS to severe sepsis ($n = 82$ and 103) [26,30], and trauma ($n = 72$, 94 and 90) [24,25,27]. The retrospective studies included patients predominately with medical diagnoses ($n = 44$ and 92) [31,32]. None of these trials included burn patients. The PCT assay used in the prospective studies were chemiluminescence [25], PCT-Q (B.R.A.H.M.S. Diagnostica, Berlin, Germany) semi-quantitative immune chromatographic assay [29], LUMItest® PCT (B.R.A.H.M.S. Diagnostica, Berlin, Germany) immune luminometric assay [26,27], and Kryptor® (B.R.A.H.M.S. Aktiengesellschaft, Germany) illuminoflourescent assay [30]; the retrospective studies used the Kryptor® assay [31,32]. Comparison between the various PCT assays has not been performed and thus similarity of threshold levels is only presumed.

Quality assessment revealed all studies used the same credible reference standard for diagnosis of sepsis [6] that was interpreted independently of the PCT screening test; reliable PCT assays were used; and when applicable, indeterminate results were reasonably interpreted. The spectrum of included patients ranged from moderate to strong, with most studies including septic and non-septic subjects, and sample sizes were also moderate to strong. Therefore, it is concluded that the rating of the included studies is of good quality.

The study by Svoboda et al. [24] randomized trauma patients ($n = 72$) to a standard treatment arm or an experimental arm ($n = 38$) in which aggressive treatment for presumed sepsis was guided when PCT test value was >2 ng/mL. The outcomes of organ failure ($8 \pm 3$ vs. $9 \pm 3$; $p = 0.06$), ICU length of stay ($16 \pm 7$ vs. $19 \pm 9$; $p = 0.09$) or ventilator days ($10 \pm 8$ vs. $14 \pm 9$; $p = 0.08$) did not reach statistical significance, perhaps due to an underpowered study. Thresholds for detection of sepsis were identified in other prospective studies as PCT value of 2 ng/mL (sensitivity of 94.7% and specificity of 78.1%) [29],and PCT value >2 ng/mL (using PCT-Q semi-quantitative test, sensitivity of 93.9% and specificity of 87.2%, with AUC 0.916) [26]. Ruiz-Alvarez et al. found a PCT level >0.32 ng/mL associated with infection with an adjusted OR of 3.8 (95% CI, 1.2–11.8) [30]. The retrospective work by Charles et al. demonstrated a higher peak PCT value when gram-negative organisms were identified than gram-positive, PCT > 16 ng/mL yielded a 83% positive predictive value (PPV) and a 74% negative predictive value (NPV) [32]; and candidemia was associated with a significantly lower value than bacterial infectious agents; values >5.5 ng/mL were more predictive of bacterial organisms with a 100% NPV and 65.2% PPV than for candidemia-related sepsis [31]. Collectively, the

**Table 2 – Characteristics of included studies (non-burn population).**

| Study | Country | Study design | Purpose of study | Sample | PCT assay | Primary findings | Conclusions | Overall findings |
|---|---|---|---|---|---|---|---|---|
| Svoboda et al. [24] | Czech Republic | RCT | Evaluate if PCT-guided diagnostic and therapeutic strategy leads to better outcome for severe sepsis after polytrauma or severe sepsis | $n$ = 72 (PCT $n$ = 38, control = 34) EXPERIMENTAL: PCT-guided treatment, PCT > 2 change ABX, <2 US/CT. CONTROL: standard evaluation by surgeon | PCT-Q (correlated with LUMItest $r = 0.93$, $p < .001$) | Experimental vs. Control: Mortality PCT 26% vs. 38%, $p = 0.28$; SOFA PCT 7.9 ± 2.8 vs. 9.3 ± 1, $p = 0.6$; ICU days PCT 16.1 ± 6.9 vs. 19.4 ± 8.9, $p = 0.09$; ventilator days PCT 10.3 ± 7.8 vs. 13.9 ± 9.4, $p = 0.08$ | Clear tendency to decrease extent of MOF when decisions based on PCT levels. PCT a marker for discriminating groups of severe sepsis patients | (+) |
| Castelli et al. [25] | Italy | Prospective observational | Diagnostic value of PCT and CRP in sepsis after trauma; secondary aim - prognostic value of PCT for severity of injury, organ dysfunction, sepsis | n-94; 76 multi-trauma, 18 TBI | PCT (chemiluminescence test) | PCT peak first day after trauma (66%), 2nd day (25%). Dramatic increase in PCT w/ onset of sepsis vs. day prior (3.32 vs. 0.85, $p < 0.001$), no change in CRP (175 vs. 135, $p$ NS) | PCT plasma reinduction marks possible septic complications during SIRS after trauma; high admission PCT indicates increased septic complication risk | (+) |
| Oh et al. [29] | Korea | Prospective observational cohort | Evaluate usefulness of semi-quantitative PCT-Q as guideline for starting ABX for sepsis in ED | ED, 80 recruited, $n$ = 33 with sepsis/septic shock | PCT-Q | PCT cut-off level 2.0 sensitivity 93.9% specificity 87.2%, AUC 0.916 | PCT probably a fast, useful method for detecting severe sepsis in ED, useful as guideline for ABX treatment | (+) |
| Ruiz-Alvarez et al. [30] | Spain | Prospective observational | Evaluate diagnostic and prognostic value of PCT as marker of infection in ICU compared to CRP and complement proteins | 103 patients with suspected sepsis (non-infected = 25, sepsis = 20, severe sepsis = 11, septic shock = 47) | Kryptor | PCT better positive likelihood ratio than CRP -2.2 vs. 1.1; SOFA highest discriminatory value 0.82, PCT 0.81; OR PCT 3.8, SOFA 5.3; PCT cut-off for sepsis 0.32, CRP 96.5 | Diagnostic accuracy PCT higher than CRP and complement PRTN; PCT combined with SOFA useful for diagnosing infection | (+) |
| Endo et al. [26] | Japan | Prospective observational | Differentiate sepsis vs. severe sepsis | $n$ = 82, no SIRS = 20, SIRS = 9, sepsis = 34, severe sepsis = 19 | LUMItest | PCT severe sepsis 36.1, sepsis 0.6, SIRS 0.0, no SIRS 0.2; Sensitivity PCT >2 94.7% specificity 78.1% | PCT may be useful for aiding diagnosis of sepsis and discriminating between sepsis and severe sepsis | (+) |
| Meisner et al. [27] | Germany | Prospective case control | Describe the time course of PCT and CRP induction in pts with trauma | $n$ = 90 adult trauma | LUMItest | PCT peak in 1-2 days, with rapid decline; CRP increased with slower decline; Sepsis, infection, blood tx, increased ICU, poor outcome with PCT >1, no correlation with increased CRP; Initial PCT 3 vs. 0.57 ($p < 0.001$) associated with subsequent sepsis | PCT provides more information than CRP after trauma; PCT returns to baseline more quickly, making subsequent increases a more valid predictor of sepsis | (+) |

**Table 2** (*Continued*)

| Study | Country | Study design | Purpose of study | Sample | PCT assay | Primary findings | Conclusions | Overall findings |
|---|---|---|---|---|---|---|---|---|
| Charles et al. [32] | France | Retrospective | Determine difference in magnitude of PCT according to pathogen present (gm−/gm+) at onset of bacteremia | 97 bacteremic episodes in 92 patients. 89.6% medical dx. Gm− $n = 52$, Gm+ $n = 45$ | Kryptor | PCT higher with Gm−, median 71.3 vs. 16.9, $p = 0.003$; PCT elevation independently associated with Gm−, OR 4.17, $p < 0.001$; PCT differentiates Gm− vs. Gm+; AUC .79, sensitivity 75%, specificity 82%, NPV 74%, cut off PCT 16 | Gm− bacteremia could be associated with higher PCT values than those found in Gm+ for septic patient; Could direct choice of ABX | (+) |
| Charles et al. [31] | France | Retrospective | PCT value in non-neutropenic critically ill patients with sepsis related to Candida spp. BSIs | BSI episodes = 50, $n = 44$; 15 candidemia episodes, $n = 11$; 70% pts medical, 30% surgical | Kryptor | PCT higher in bacteremia vs. Candidemia, low PCT independent predictor of Candidemia (0.96). PCT > 5.5 NPV 100%, PPV 65.2% | High PCT value in critically ill non-neutropenic patient with clinical sepsis unlikely in setting of Candidemia | (+) |

ABX: antibiotic; AUC: area under the receiver operating curve; BSI: blood stream infection; CRP: C-reactive protein; CT: computed tomography scan; ED: Emergency Department; gm−: gram-negative bacteria; gm+: gram-positive bacteria; NPV: negative predictive value; OR: odds ratio; PCT: procalcitonin; PPV: positive predictive value; SIRS: systemic inflammatory response syndrome; SOFA: sequential organ failure score; tx: transfusion; US: ultrasound. PCT levels reported in ng/mL.

findings from these clinical trials found PCT to be a useful marker of sepsis as levels >2 ng/mL were positively associated with severe bacterial infection in the majority of the studies.

### 3.3. *Burn specific clinical trials*

Six clinical trials conducted on burn injured patients were retrieved: five prospective observational studies [16–19,28] and one retrospective review [33]. One study focused on a pediatric population ($n = 20$; age range 0.9–11.3 years, mean 6.0 years) with a mean total body surface area percent (TBSA %) of $49.5 \pm 4.5\%$ (range 20–94%) with full-thickness injury of $40.5 \pm 5.8\%$ (range 0–94%) [28]. The other prospective studies included a total of 155 (mean $39 \pm 9.9$; age range 25–60) adult burn patients; the retrospective study enrolled 19 patients. Mean TBSA % in the adult prospective studies was 48.5% (range 41.4–62%) [16–19], one study used a control group (mean TBSA $58 \pm 17\%$) [16] and the other used subjects as their own controls. Mean TBSA was 32% for all patients ($n = 19$) and 41% for septic patients ($n = 9$) in the retrospective study (*p* value not reported) [33]. Only the pediatric study [28] was conducted in the United States, the other trials were performed in European countries. The LUMItest® PCT assay was used for five studies [17–19,28,33] and the PCT-Q semi-quantitative assay was used exclusively in one study [16] and correlated with the LUMItest® in one study [17].

Assessment of the quality of included studies demonstrated use of a relevant and reliable screening test for all studies, and appropriate discussion of indeterminate results as applicable. As previously discussed, use of a reference standard for detection of sepsis in general ICU population is not appropriate for a burn patient who has baseline SIRS, so caution must be taken when interpreting results for the studies that did not report additional criteria for sepsis diagnosis other than the ACCP/SCCM guidelines [6,16–18]. One study [33] used supplementary indicators such as CRP, lactate level, and vasopressor or fluid requirement for determination of sepsis. The Baltimore Sepsis Scale [34] was used in one study [19], and although this scale was developed for use in a burn population and is fairly robust, incorporating 13 parameters associated with sepsis, it has not been used prospectively or validated. Finally, the study by Neely et al. [28] did not use any standardized measure of sepsis, but instead used criteria developed by the researchers, based on serum CRP values [35]. Therefore, although the reference standard was used in all cases independently from the screening PCT test, overall the quality rating for reference standards used for this burn specific population is fair at best. All of these studies used a population with and without sepsis but included a relatively small homogenous burn population, so a moderate degree of confidence can be ascribed to the sampling method. Overall, the quality of the burn specific studies included in this review can be given a fair quality assessment. The level of evidence for all included burn studies is "C" (descriptive studies) [22].

Various cut-off levels of PCT were noted among the burn specific studies, from 0.5 ng/mL (sensitivity 100% and specificity 89.8% for diagnosis of sepsis) [16] and 0.53 ng/mL (sensitivity 42.4% and 88.8%) [17] to 3.0 ng/mL threshold associated with septic complications [19]. The retrospective

**Table 3 – Characteristics of included studies of burn patients.**

| Study | Country | Study design | Purpose of study | Sample | PCT assay | Primary findings | Conclusions | Overall findings |
|---|---|---|---|---|---|---|---|---|
| Barati et al. [16] | Iran | Prospective observational | Compare inflammatory markers for diagnosis of sepsis in burn (PCT, CRP, ESR, WBC) | Burn $n = 60$ (sepsis = 30, 62 ± 21% TBSA, non-septic = 30, 58+-17% TBSA) | PCT-Q | PCT with/without sepsis 8.45 ± 7.8 vs. 0.5 ± 1.0, $p < .001$. No difference in CRP, ESR, WBC; PCT cut-off 0.5 ($p < 0.001$) sensitivity 100%, specificity 89.8% | PCT highly efficient lab parameter for diagnosis of severe septic complications after burn, but not CRP, ESR, WBC | (+) |
| Bargues et al. [17] | France | Prospective observational | Analyze PCT as marker of sepsis in burn pts; Compare LUMItest and PCT-Q | Burn $n = 25$, 40 ± 17 TBSA% | LUMItest & PCT-Q | AUC no different PCT, CRP, WBC; PCT cut-off 0.53, sensitivity 42.4, specificity 88.8. CRP cut-off 102 mg/l, sensitivity 79.5 specificity 60.3 | PCT not superior to CRP/WBC as marker of sepsis in burn patients | (–) |
| Lavrentieva et al. [18,36] | Greece | Prospective observational | Estimate diagnostic value of PCT, CRP, WBC, temp as marker of sepsis in BICU | $n = 43$ burn ICU, 41.4 ± 22%TBSA | LUMItest | PCT sepsis vs. SIRS 11.8 ± 15.8 vs. 0.63 ± 0.043, $p < .001$; AUC 0.975, $p = 0.002$, PCT has + discriminatory power; CRP, WBC, temp no difference; PCT cut-off 1.5–2.0 | Serum PCT useful as early indicator of septic complications in patients with severe burn injury | (+) |
| Neely et al. [28] | USA | Prospective observational | Determine if daily monitoring of PCT improved early diagnosis of sepsis | $n = 20$ pediatric, 49.5 ± 4.5%TBSA (26 septic, 36 non-septic episodes) | LUMItest | PCT sensitivity 42%, specificity 67%, efficiency 57%; Surgeon identified sepsis 0.8 days earlier than PCT change using CRP and platelet count | PCT not as effective as CRP and/or platelet count in early detection of sepsis in burned children | (–) |
| von Heimburg et al. [19] | Germany | Prospective observational | Determine PCT levels and correlate levels with inhal injury, TBSA, outcome, infection in severely burned | $n = 27$, 51%TBSA, 3 electrical burn | LUMItest | PCT higher septic vs non-septic 49.8 ± 76.9 vs. 2.3 ± 3.7, $p < .005$; Cut off value 3.0 reliable to indicate severe bacteremia/fungal infection | Individual course of PCT in one patient is more important than absolute values; PCT useful diagnostic parameter | (+) |
| Sachse et al. [33] | Germany | Retrospective | Temporal analysis of PCT to determine diagnostic and prognostic value of this parameter in burn pts | $n = 19$ adult burn; 32% TBSA. $N = 9$ septic, 41% TBSA | LUMItest | Septic pts PCT > after day 7; no difference in CRP. Rise in PCT correlated to septic symptoms, increase 1.5 in 8/9 pts; PCT > 15 only noted in pts who died of septic related MOF | Individual time course of PCT of diagnostic value, efficient parameter for diagnosis of severe infection in burn pts | (+) |

AUC: area under the receiver operating curve; CRP: C-reactive protein; ESR: erythrocyte sedimentation rate; MOF: multiple organ failure; PCT: procalcitonin; TBSA: total body surface area; WBC: white blood cells. PCT levels reported in ng/mL.

report by Sachse et al. [33] describes a 1.5 ng/mL rise in daily PCT levels associated with onset of septic events. The study of pediatric burn patients found no improvement in detection of sepsis using PCT compared with CRP, the diagnostic standard of care for this center (sensitivity 42.4% and specificity 88.8%) [28]. Finally, Lavrentieva et al. [18] found the PCT cut-off level of 1.5 ng/mL to have the highest sensitivity and specificity (82% and 91.2%, respectively) when contrasted with thresholds of 2 ng/mL and 2.5 ng/mL (66.6% and 96.8% versus 66.6% and 97.6%, respectively). Von Heimburg et al. demonstrated a correlation between increasing TBSA and increasing PCT level [19]. Overall a lack of consensus exists for utility of the PCT assay to reliably detect sepsis in the burn specific population due to the contradictory findings of two [17,28] of the reviewed studies, despite positive findings for PCT use in 3 prospective [16,19,36] and one retrospective [33] study.

## 4. Discussion

Collectively, the body of available evidence supports the utility of PCT assay as an adjunct to sepsis diagnosis in the critically ill population. The meta-analysis by Tang et al. [21] was unable to support the clinical value of this test. However, the area under the ROC curve for the pooled studies ($n = 18$) was 0.78 (95% CI 0.73–0.83), with a diagnostic OR of 7.79 (95% CI 5.86–10.35). These authors do suggest the additive value of the PCT assay to contribute to clinical diagnosis of sepsis. Furthermore, although consensus is absent within the burn literature perhaps the small number of patients studied, inclusion of a pediatric study in this analysis, or the underlying metabolic complexity of the severely burned patients confounds these findings.

One primary inconsistency in the burn specific studies is reliance on a reference standard for diagnosis sepsis intended for a different ICU population; the ACCP/SCCM guidelines [6] describe SIRS, the metabolic baseline for the burn patient [7]. Use of an accepted standard for sepsis diagnosis for burn patients is necessary to guide any studies directed toward this unique population. The study by Barque et al. [17] relied on the ACCP/SCCM sepsis guidelines which identify SIRS, yet the population studied was comprised of predominately respiratory (18/47 subjects) and wound infections (15/47 subjects) which are prone to improper diagnosis due to high rates of colonization. Coupled with the conservative PCT cutoff level of 0.53 ng/mL for sepsis determination this study may have utilized a population with mild to moderate infections not representative of severely ill burn patients [17].

The pediatric population studied by Neely et al. [28] may suffer from lack of an objective standard for sepsis diagnosis; this study relied on the subjective determination of sepsis by a burn surgeon. Furthermore, a dramatic rise in PCT of 5 ng/mL was identified as necessary for diagnosis of sepsis but median (25%, 75% quartile) PCT for septic and non-septic patients were reported as 6.7 (3.7, 31.2) vs. 2.1 (1.3, 5.7) ($p < 0.002$) respectively. Perhaps the 5 ng/mL threshold was too ambitious considering the moderate PCT levels for the septic pediatric subjects. Finally, Neely et al. concluded with the suggestion that had CRP not been the burn center's standard of care for diagnosis of sepsis use of PCT would likely have decreased time to treatment. Thus, it may prove to be useful to utilize CRP or PCT interchangeably in the burn population to expedite sepsis treatment.

It would seem the conclusion from this review of PCT assay effectiveness in burns to be a promising adjunct to clinical management of septic patients. However, reliance on observational and retrospective reviews to guide clinical care is tenuous at best. Well controlled clinical trials, preferably conducted in multiple centers will guide future knowledge related to how PCT assay contributes to early identification and treatment of burn sepsis. The expectation of such trials is use of the ABA sepsis criteria [7] to identify severe infection, with validation of these parameters in a prospective manner. As noted, these guidelines are currently the best available but were formulated by consensus and require robust substantiation.

Improved sensitivity and specificity for prediction of sepsis may be conferred when assay results are coupled with clinical indicators in a systematic manner. In the meantime, clinical care would be supported with routine measurement of PCT on a daily basis to detect acute changes in the baseline level for patients at high risk for sepsis. Such an assay requires a minimal blood sample, the equivalent of routine chemistry or hematological studies, and due to recent technological advances this assay will soon become cost-effective. Unfortunately, at many institutions PCT is processed elsewhere, taking several days for quantification with a cost of several hundred dollars. Certainly this constraint eliminates the utility of this screening assay to detect daily changes in PCT levels to initiate expeditious treatment. Federal Drug Administration approval for the United States is pending for a simple semi-quantitative test using a dip-stick and colorimetric results that will be practical and cost-effective. This test, PCT-Q has been used in the emergency department and demonstrated to be a fast and effective method of initiating antibiotic therapy in that setting [29]. Svoboda et al. reported a correlation of $r = 0.92$ of the PCT-Q with the quantitative PCT LUMItest® [24]. The ranges of the PCT-Q are clinically relevant to the thresholds associated with clinically significant degrees of infection identified in the literature for local infection, systemic infection (sepsis) and severe sepsis of 0.5, 2, and 10 ng/mL respectively [29].

Other forthcoming technology will make availability of PCT assay practical using devices such as Theranos™ (Theranos, Inc, Palo Alto, CA) point of care technology, a customizable device for multiple assays that includes the PCT test. Modules compatible with widely used core laboratory equipment to provide on-site quantitative PCT assay are available, making routine screening of PCT a practical and clinically useful adjunct to our current diagnosis and management of burn sepsis.

Fortunately, routine screening of PCT conveys no additional patient risk, as this test is non-invasive, requiring minimal phlebotomy, and will serve as an adjunct to routine clinical decision making. A large prospective multi-center RCT is underway in Europe (planned enrollment $n = 1000$ ICU patients) to determine the efficacy of guiding antibiotic therapy for infection using daily PCT levels (The Procalcitonin and Survival Study - PASS) [37], powered to determine mortality benefit of PCT guided therapy. A previous multi-

center RCT conducted in the emergency department setting (The ProHOSP Randomized Controlled Trial) determined PCT guided antibiotic therapy for lower respiratory tract infections reduced antibiotic exposure and associated adverse effects with no increase of adverse outcomes [38]. These studies support the premise that routine monitoring of PCT in the critically ill confers minimal risk, and promises benefits of reduced exposure to antibiotic therapy, directed antibiotic therapy, and the potential for reduction in mortality associated with infection. These areas for future research should be extended to the burn community, where risk of death from sepsis is great [1].

Limits of this review include reliance on a single reviewer for the articles selected for inclusion, inconsistent findings, and use of various PCT quantification techniques among the various research studies. As the overwhelming majority of included studies were performed outside of the United States the conclusions related to applicability to an American population may differ based on practice differences and available technology. Perhaps the wide-spread availability of inexpensive, in-house PCT assay will promote greater use of this diagnostic tool.

In conclusion, PCT assay can be a helpful adjunct to clinical diagnosis of sepsis and holds promise as a method for reducing antibiotic exposure in the critically ill patient. Further research will elucidate the value of PCT guided diagnosis and therapy on outcomes such as hospital stay and mortality. Availability of an inexpensive and rapid assay remains the central obstacle to routine use of this test. Once the assay is incorporated into routine care in a large number of U.S. burn centers multi-center randomized trials will provide evidence of benefit in guiding antibiotic therapy and survival outcomes for this vulnerable population.

## Acknowledgements

Clara Fowler, Librarian, MD Anderson Cancer Center; CPT Kelly Wilhelms, Chief, Core Laboratory, Brooke Army Medical Center.

## REFERENCES

[1] Fitzwater J, Purdue GF, Hunt JL, O'Keefe GE. The risk factors and time course of sepsis and organ dysfunction after burn trauma. J Trauma 2003;54(5):959–66.

[2] D'Avignon L, Hogan B, Murray C, Loo F, Hospenthal D, Cancio L, et al. Contribution of bacterial and viral infections attributable to mortality in patients with severe burns: an autopsy series. Burns 2010. doi:10.1016/j.burns.2009.11.007.

[3] Sharma BR, Harish D, Singh VP, Bangar S. Septicemia as a cause of death in burns: an autopsy study. Burns 2006;32(5):545–9.

[4] Finnerty CC, Herndon DN, Przkora R, Pereira CT, Oliveira HM, Queiroz DM, et al. Cytokine expression profile over time in severely burned pediatric patients. Shock 2006;26(1):13–9.

[5] Murray CK, Hoffmaster RM, Schmit DR, Hospenthal DR, Ward JA, Cancio LC, et al. Evaluation of white blood cell count, neutrophil percentage, and elevated temperatures as predictors of bloodstream infection in burn patients. Arch Surg 2007;142(7):639–42.

[6] Levy MM, Fink MP, Marshall JC, Abraham E, Angus D, Cook DJ, et al. 2001 SCCM/ESICM/ACCP/ATS/SIS International sepsis definitions conference. Crit Care Med 2003;31:1250–6.

[7] Greenhalgh DG, Saffle JR, Holmes JH, Gamelli RL, Palmieri TL, Horton JW, et al. American Burn Association consensus conference to define sepsis and infection in burns. J Burn Care Res 2007;28:776–90.

[8] Lodise Jr TP, Patel N, Kwa A, Graves J, Furuno JP, Graffunder E, et al. Predictors of 30-day mortality among patients with *Pseudomonas aeruginosa* bloodstream infections: impact of delayed appropriate antibiotic selection. Antimicrob Agents Chemother 2007;51(10):3510–5.

[9] Schneider HG, Lam QT. Procalcitonin for the clinical laboratory: a review. Pathology 2007;39(4):383–90.

[10] Wanner GA, Keel M, Steckholzer U, Beier W, Stocker R, Ertel W. Relationship between procalcitonin plasma levels and severity of injury, sepsis, organ failure, and mortality in injured patients. Crit Care Med 2000;28(4):950–7.

[11] Gabay C, Kushner I. Acute-phase proteins and other systemic responses to inflammation. N Engl J Med 1999;340(6):448–54.

[12] Harbarth S, Holeckova K, Froidevaux C, Pittet D, Ricou B, Grau GE, et al. Diagnostic value of procalcitonin, interleukin-6, and interleukin-8 in critically ill patients admitted with suspected sepsis. Am J Respir Crit Care Med 2001;164(3):396–402.

[13] Jones AE, Fiechtl JF, Brown MD, Ballew JJ, Kline JA. Procalcitonin test in the diagnosis of bacteremia: a meta-analysis. Ann Emerg Med 2007;50(1):34–41.

[14] Simon L, Gauvin F, Amre DK, Saint-Louis P, Lacroix J. Serum procalcitonin and C-reactive protein levels as markers of bacterial infection: a systematic review and meta-analysis. Clin Infect Dis 2004;39(2):206–17.

[15] Uzzan B, Cohen R, Nicolas P, Cucherat M, Perret GY. Procalcitonin as a diagnostic test for sepsis in critically ill adults and after surgery or trauma: a systematic review and meta-analysis. Crit Care Med 2006;34(7):1996–2003.

[16] Barati M, Alinejad F, Bahar MA, Tabrisi MS, Shamshiri AR, Bodouhi NO, et al. Comparison of WBC, ESR, CRP and PCT serum levels in septic and non-septic burn cases. Burns 2008;34(6):770–4.

[17] Bargues L, Chancerelle Y, Catineau J, Jault P, Carsin H. Evaluation of serum procalcitonin concentration in the ICU following severe burn. Burns 2007;33:860–4.

[18] Lavrentieva A, Kontakiotis T, Lazaridis L, Tsotsolis N, Koumis J, Kyriazis G, et al. Inflammatory markers in patients with severe burn injury: what is the best indicator of sepsis? Burns 2007;33:189–94.

[19] von Heimburg D, Steighorst W, Khorram-Sefat R, Pallua N. Procalcitonin—a sepsis parameter in severe burn injuries. Burns 1998;24:745–50.

[20] Sakr Y, Sponholz C, Tuche F, Brunkhorst F, Reinhart K. The role of procalcitonin in febrile neutropenic patients: review of the literature. Infection 2008;36(5):396–407.

[21] Tang BM, Eslick GD, Craig JC, McLean AS. Accuracy of procalcitonin for sepsis diagnosis in critically ill patients: systematic review and meta-analysis. Lancet Infect Dis 2007;7(3):210–7.

[22] Armola RR, Bourgault AM, Halm MA, Board RM, Bucher L, Harrington L, et al. Upgrading the American Association of Critical-Care Nurses' evidence-leveling hierarchy. Am J Crit Care 2009;18(5):405–9.

[23] US Preventive Services Task Force Evidence Syntheses. Appendix 2: US Preventive Services Task Force quality rating criteria: diagnostic accuracy studies. [December 4, 2009]; Available from: http://www.ncbi.nlm.nih.gov/bookshelf/br.fcgi?book=hsevidsyn&part=A52999.

[24] Svoboda P, Kantorova I, Scheer P, Radvanova J, Radvan M. Can procalcitonin help us in timing of re-intervention in septic patients after multiple trauma or major surgery? Hepatogastroenter 2007;54(74):359–63.
[25] Castelli GP, Pognani C, Cita M, Paladini R. Procalcitonin as a prognostic and diagnostic tool for septic complications after major trauma. Crit Care Med 2009;37(6):1845–9.
[26] Endo S, Aikawa N, Fujishima S, Sekine I, Kogawa K, Yamamoto Y, et al. Usefulness of procalcitonin serum level for the discrimination of severe sepsis from sepsis: a multicenter prospective study. J Infect Chemother 2008;14(3):244–9.
[27] Meisner M, Adina H, Schmidt J. Correlation of procalcitonin and C-reactive protein to inflammation, complications, and outcome during the intensive care unit course of multiple-trauma patients. Crit Care 2006;10(1):R1.
[28] Neely AN, Fowler LA, Kagan RJ, Warden GD. Procalcitonin in pediatric burn patients: an early indicator of sepsis? J Burn Care Rehabil 2004;25(1):76–80.
[29] Oh JS, Kim SU, Oh YM, Choe SM, Choe GH, Choe SP, et al. The usefulness of the semiquantitative procalcitonin test kit as a guideline for starting antibiotic administration. Am J Emerg Med 2009;27(7):859–63.
[30] Ruiz-Alvarez MJ, Garcia-Valdecasas S, De Pablo R, Sanchez Garcia M, Coca C, Groeneveld TW, et al. Diagnostic efficacy and prognostic value of serum procalcitonin concentration in patients with suspected sepsis. J Int Care Med 2009;24(1):63–71.
[31] Charles PE, Dalle F, Aho S, Quenot JP, Doise JM, Aube H, et al. Serum procalcitonin measurement contribution to the early diagnosis of candidemia in critically ill patients. Int Care Med 2006;32(10):1577–83.
[32] Charles PE, Ladoire S, Aho S, Quenot JP, Doise JM, Prin S, et al. Serum procalcitonin elevation in critically ill patients at the onset of bacteremia caused by either Gram negative or Gram positive bacteria. BMC Infect Dis 2008;8:38.
[33] Sachse C, Machens HG, Felmerer G, Berger A, Henkel E. Procalcitonin as a marker for the early diagnosis of severe infection after thermal injury. J Burn Care Rehabil 1999;20(5):354–60.
[34] Meek M, Munster AM, Winchurch RA, Dickerson C. The Baltimore Sepsis Scale: measurement of sepsis in patients with burns using a new scoring system. J Burn Care Rehabil 1991;12(6):564–8.
[35] Neely AN, Smith WL, Warden GD. Efficacy of a rise in C-reactive protein serum levels as an early indicator of sepsis in burned children. J Burn Care Rehabil 1998;19(2):102–5.
[36] Lavrentieva A, Kontakiotis T, Lazaridis L, Tsotsolis N, Koumis J, Kyriazis G, et al. Inflammatory markers in patients with severe burn injury. What is the best indicator of sepsis? Burns 2007;33(2):189–94.
[37] Jensen JU, Lundgren B, Hein L, Mohr T, Petersen PL, Andersen LH, et al. The Procalcitonin And Survival Study (PASS)—a randomised multi-center investigator-initiated trial to investigate whether daily measurements biomarker Procalcitonin and pro-active diagnostic and therapeutic responses to abnormal Procalcitonin levels, can improve survival in intensive care unit patients. Calculated sample size (target population): 1000 patients. BMC Infect Dis 2008;8:91.
[38] Schuetz P, Christ-Crain M, Thomann R, Falconnier C, Wolbers M, Widmer I, et al. Effect of procalcitonin-based guidelines vs standard guidelines on antibiotic use in lower respiratory tract infections: the ProHOSP randomized controlled trial. JAMA 2009;302(10):1059–66.
